# Macrofungi as Medicinal Resources in Uzbekistan: Biodiversity, Ethnomycology, and Ethnomedicinal Practices

**DOI:** 10.3390/jof9090922

**Published:** 2023-09-13

**Authors:** Yusufjon Gafforov, Milena Rašeta, Sylvie Rapior, Manzura Yarasheva, Xuewei Wang, Liwei Zhou, Wan Abd Al Qadr Imad Wan-Mohtar, Muhammad Zafar, Young Woon Lim, Mengcen Wang, Bekhzod Abdullaev, Rainer W. Bussmann, Gokhan Zengin, Jiajia Chen

**Affiliations:** 1 New Uzbekistan University, Tashkent 100007, Uzbekistan; 2 Central Asian University, Tashkent 111221, Uzbekistan; 3 Mycology Laboratory, Institute of Botany, Academy of Sciences of Republic of Uzbekistan, Tashkent 100125, Uzbekistan; 4 State Key Laboratory of Mycology, Institute of Microbiology, Chinese Academy of Sciences, Beijing 100101, China; 5 Department of Chemistry, Biochemistry and Environmental Protection, Faculty of Sciences, University of Novi Sad, Trg Dositeja Obradovića 3, 21000 Novi Sad, Serbia; 6 CEFE, CNRS, University of Montpellier, EPHE, IRD, 15 Avenue Charles Flahault, CS 14491, CEDEX 5, 34093 Montpellier, France; 7 Laboratory of Botany, Phytochemistry and Mycology, Faculty of Pharmacy, 15 Avenue Charles Flahault, CS 14491, CEDEX 5, 34093 Montpellier, France; 8 Tashkent International University of Education, Tashkent 100207, Uzbekistan; 9 University of Chinese Academy of Sciences, Beijing 101408, China; 10 Functional Omics and Bioprocess Development Laboratory, Institute of Biological Sciences, Faculty of Science, University Malaya, Kuala Lumpur 50603, Malaysia; 11 Department of Plant Sciences, Quaid-i-Azam University, Islamabad 45320, Pakistan; 12 School of Biological Sciences, Institute of Microbiology, Seoul National University, Seoul 08826, Republic of Korea; 13 State Key Laboratory of Rice Biology, Ministry of Agricultural and Rural Affairs Laboratory of Molecular Biology of Crop Pathogens and Insects, Zhejiang University, Hangzhou 310058, China; 14 Department of Ethnobotany, State Museum of Natural History, 76133 Karlsruhe, Germany; rainer.bussmann@iliauni.edu.ge; 15 Department of Ethnobotany, Institute of Botany and Bakuriani Alpine Botanical Garden, Ilia State University, Botanical Street 1, 0105 Tbilisi, Georgia; 16 Department of Biology, Science Faculty, Selçuk University, Konya 42130, Turkey; 17 College of Landscape Architecture, Jiangsu Vocational College of Agriculture and Forestry, Zhenjiang 212400, China

**Keywords:** *Basidiomycetes*, distribution, ethnomedicine, medicinal uses, molecular phylogeny, taxonomic diversity, substrate preferences

## Abstract

Interest in edible and medicinal macrofungi is millennial in terms of their uses in health and food products in Central Asia, while interest in inedible and medicinal macrofungi has grown in popularity in recent years. Edible and inedible medicinal basidiomycetes were collected during field surveys from different regions of Uzbekistan. The morphological characters and similarity assessment of rDNA-Internal Transcribed Spacer sequence data were used to measure diversity and habitat associations. A number of 17 species of medicinal macrofungi of ethnomycological and medicinal interest was found associated with 23 species of trees and shrubs belonging to 11 families and 14 genera. *Polyporaceae* and *Hymenochaetaceae* were represented by the highest number of species followed by *Ganodermataceae*, *Fomitopsidaceae*, *Auriculariaceae*, *Cerrenaceae*, *Grifolaceae*, *Phanerochaetaceae*, *Laetiporaceae*, *Schizophyllaceae*, and *Stereaceae*. The highest number of medicinal basidiomycete species was reported in the following host genera: *Acer*, *Betula*, *Celtis*, *Crataegus*, *Juglans*, *Juniperus*, *Lonicera*, *Malus*, *Morus*, *Platanus*, *Populus*, *Prunus*, *Quercus*, and *Salix*. An updated list of edible and inedible medicinal mushrooms identified in Uzbekistan, their morphological characteristics, and phylogenetic placement are given for the first time. Information is provided on their uses in traditional and modern medicine. Their bioactive compounds and extracts can be applied as medicines, as well as food and cosmetic ingredients.

## 1. Introduction

Ethnomycology, an interdisciplinary field that explores the cultural and traditional uses of fungi, plays a pivotal role in documenting, presenting, and preserving the rich and diverse knowledge associated with the utilization of mushrooms and other fungal organisms in biology and ethnomedicine. Building upon this foundation, the ethnomycological study of the selected macrofungal species, including esteemed genera such as *Fomes*, *Inonotus*, *Ganoderma*, *Phellinus*, *Trametes*, and other macrofungal groups, has revealed their extensive utilization in health practices for millennia [1,2,3,4,5]. Notably, the opulent and valuable documentation of fungal species uses in folk medicine has particularly flourished in East Asia, encompassing China, Japan, and Korea [5,6].

Medicinal fungi are currently studied by many ethnomycologists and medical researchers. In particular, and most importantly for modern medicine, macrofungi are a huge source of triterpene derivatives [7,8], polysaccharides, and polysaccharide-protein complexes with anti-cancer and immune-stimulating properties [9,10,11,12,13,14,15,16]. Scientists are currently focusing on macrofungi, specifically a group of poroids that have antitumor effects and help boost the immune system. Today, polysaccharides are the main constituents of some medicines and nutritional supplements well-known worldwide including in Southeast Asia [17,18,19,20,21].

Our investigation aimed to assess medicinal macrofungi in Uzbekistan and document the uses of specific mushroom species in the study area. Despite mycological research in Uzbekistan dating back to the 20th century, medicinal macrofungi are largely overlooked [22,23,24,25,26,27,28,29,30,31]. Limited information exists on the uses of macrofungi in Central Asian folk medicine, and their biodiversity remains poorly explored [32,33,34,35,36,37]. Recent DNA sequencing efforts revealed previously unknown and scientifically novel species of ascomycete and basidiomycete fungi in the Uzbekistan region [27,28,30,32,36,38,39,40,41,42,43,44].

In addition, the aim of this study was to examine the biodiversity and systematic composition, as well as establish a modern list of medicinal mushrooms in Uzbekistan. We also intend to obtain data on the applications of particular species in both traditional and modern medical uses and to design a database on ethnomedicinal mushrooms in Uzbekistan.

## 2. Materials and Methods

### 2.1. Study Area

**Figure 1:**
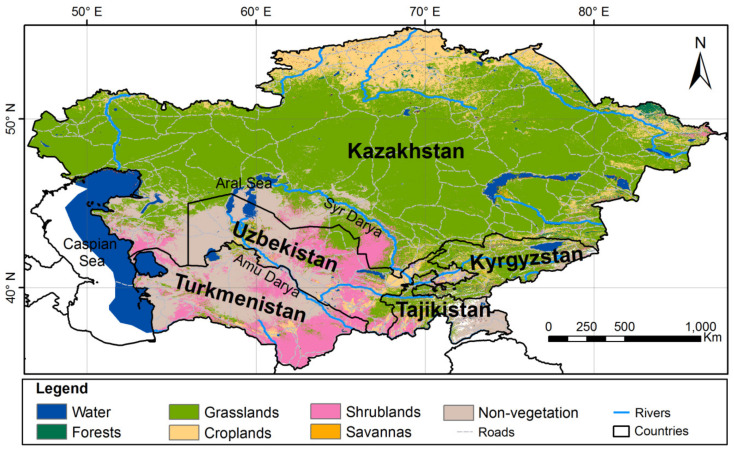
Map of Uzbekistan [32].

Uzbekistan, located in Central Asia extends from the foothills of the Tian Shan and Pamir mountains in the east to west of the Aral Sea. Uzbekistan borders Kazakhstan to the north, Kyrgyzstan and Tajikistan to the east and southeast, Turkmenistan to the west, and Afghanistan to the south (Figure 1). The country covers 447,400 km^2^ (172,742 sq miles) and has a population of about 35 million. Uzbekistan is divided into 12 provinces and one autonomous republic with desert and mountainous areas, where continental climate mainly prevails. It is one of the most diverse regions in the world with respect to both fauna and flora; it is considered a remarkable collection of many species of medicinal plants [45,46].

### 2.2. Collection and Preservation of Medicinal Macrofungi Samples

This study is based on fresh basidiomata of medicinal basidiomycetes collected between 2021 and 2022 during field surveys in the provinces of Andijan, Fergana, Jizzakk, Namangan, Samarkand, Tashkent, and in the botanical garden of the city of Tashkent in Uzbekistan (Table 1). Dried specimens were used in the laboratory of mycology from the Institute of Botany to determine the taxonomic composition of species with references from various books and monographs. The collected samples were deposited at TASM (Tashkent Mycological Herbarium), the Institute of Botany of the Academy of Science of Uzbekistan after morphological study. Fungarium acronyms used in the paper are from *Index Herbariorum* [47].

### 2.3. Identification of the Medicinal Macrofungi

#### 2.3.1. Morphological Observations

Morphological characters were described based on fresh and dried fruiting bodies. Microscopic characters of the fruiting bodies were observed on dried specimens at a magnification up to 1000× with a Leica DM 1000 (Leica Microsystems, Wetzlar, Germany) microscope in 5% aqueous KOH plus 1% phloxine, Melzer’s reagent for amyloid or dextrinoid reactions, cotton blue in lactic acid for cyanophily, and 1% aqueous cresyl blue for metachromatism. Macromorphological characters of the fruiting bodies and hymenophores were observed under a Leica M165 FC stereomicroscope (Leica Microsystems, Wetzlar, Germany).

#### 2.3.2. DNA Extraction, Amplification, Sequencing, and Phylogenetic Analyses

From recently collected dried specimens, 5–10 mg of the fruiting body was taken and disrupted in a mixer mill (MM2, Retsch, Germany), using two iron beads of 3 mm and five beads of 1 mm diameter per sample and shaking twice at maximum speed for 15 min. Genomic DNA was extracted using the BioSprint 96 DNA Plant Kit (QIAGEN Diagnostics GmbH, Qiagen AG, Germany) on a KingFisher Flex (Thermo Fisher Scientific, Waltham, Massachusetts, United States) robot. The amplification via PCR of the internal transcribed spacer (ITS) was performed using primers ITS1F/ITS4 or ITS1/ITS4 [48,49]. Amplicons were sequenced at the Biodiversity and Climate Research Centre (BiK-F) laboratory using primers identical to those used for amplifications.

The assembled sequences obtained in this study were deposited in the NCBI GenBank (Table 2). Each sequence was compared with the reference sequences in GenBank, using a BLAST search [50]. The ITS region was used to identify each species of medicinal mushrooms in Uzbekistan. Accordingly, some ITS sequences were downloaded from GenBank and incorporated with the newly generated ITS sequences in a dataset (Table 2). The ingroup taxa belong to Agaricales, Auriculariales, Hymenochaetales, Polyporales, and Russulales, while *Coniophora arida* and *Gomphidius roseus* from Boletales were selected as outgroup taxa.

MAFFT v.7.110 [51] was chosen to align the ITS region under the “G-INS-i” option [52]. jModelTest v.2.1.10 [53,54] was used to estimate the best-fit evolutionary model of the alignment for phylogenetic analyses under the Akaike information criterion. Maximum Likelihood (ML) and Bayesian Inference (BI) algorithms were utilized for phylogenetic analyses. The ML algorithm was conducted using raxmlGUI v.8.2.12 [55] with the calculation of bootstrap (BS) replicates under the auto FC option [56]. The BI algorithm was performed using MrBayes v.3.2.7 [57]. Two independent runs were employed and each run included four chains and started from random trees. Trees were sampled every 1000th generation, and the first 25% of the sampled trees were removed, while the other 75% were retained for constructing a 50% majority consensus tree and calculating Bayesian posterior probabilities (BPPs). Chain convergence of the resulting log file was judged using Tracer v.1.7.1 [58]. The final phylogenetic tree was edited and visualized by tvBOT [59].

### 2.4. Data Collection

We obtained distribution, habitats, taxonomy, morphology, ethnobotanical data, and ethnomedicinal uses of medicinal fungal from previously published literature, i.e., research articles, monographs, and books written in Uzbek, Russian, and English in indexed and non-indexed journals using online bibliographic databases, as well as local library sources and our own materials. In addition, scientific names of medicinal fungi and their hosts were checked for potential synonyms in *Index Fungorum* and *Plants of the World Online* [60,61]. Data analysis, as well as field interviews and discussions, were conducted with forest inspectors in Uzbekistan.

**Table 2:** Voucher numbers and corresponding GenBank accession numbers of sequences used for phylogenetic analyses.

Species Name	Specimens	Host/Substrate	Origin	GenBank Acc. No.	References
*Auricularia mesenterica* (Dicks.) Pers.	YG/PS177	*Prunus vulgaris* (Mill.) Schur	Uzbekistan	OR250340	Present study
*Auricularia mesenterica*	YG 087	*Prunus armeniaca* L.	Uzbekistan	OR250341	Present study
*Auricularia mesenterica*	Haikonen 11208	Rotten angiosperm trunk	United Kingdom	KP729287	[62]
*Bjerkandera adusta* (Willd.) P. Karst.	YG 013	*Juglans regia* L.	Uzbekistan	OR250343	Present study
*Bjerkandera adusta*	YG/PS15	*Populus alba* L.	Uzbekistan	OR250342	Present study
*Bjerkandera adusta*	BRNM 771946	dead hardwood	Brazil	KT305936	[63]
*Cerrena unicolor* (Bull.) Murrill	YG 027	*Acer tataricum* subsp. *Semenovii* (Regel & Herder) A.E. Murray	Uzbekistan	OR250344	Present study
*Cerrena unicolor*	YG/PS16	*Crataegus turkestanica* Pojark.	Uzbekistan	OR250345	Present study
*Cerrena unicolor*	FD-299	Dead standing hardwood	USA	KP135304	[64]
*Fomes fomentarius* (L.) Fr.	351_YG20211004-1D	*Juglans regia*	Uzbekistan	OR250349	Present study
*Fomes fomentarius*	349_YG20210406-8D	*Malus domestica* (Suckow) Borkh.	Uzbekistan	OR250348	Present study
*Fomes fomentarius*	YG-M155	Unknown fallen wood	Uzbekistan	OR250347	Present study
*Fomes fomentarius*	YG-M200	*Betula tianschanica* Rupr.	Uzbekistan	OR250346	Present study
*Fomes fomentarius*	Ff/25	Unknown	Armenia	OL583672	[65]
*Fomes fomentarius*	YG/bot2	*Populus* sp.	Uzbekistan	MT526299	[33]
*Ganoderma adspersum* (Schulzer) Donk	YG20210406-3D	*Acer saccharum* Marshall	Uzbekistan	OR250350	Present study
*Ganoderma adspersum*	Ga-3	*Acacia* sp.	Armenia	JN588585	[66]
*Inonotus hispidus* (Bull.) P. Karst.	343_YG20210525-1D	*Juglans regia*	Uzbekistan	OR250351	Present study
*Inonotus hispidus*	JV0407/31	*Juglans* sp.	Morocco	KF446596	[67]
*Inonotus hispidus*	YG/PS148	*Malus sieversii* (Ledeb.) M. Roem.	Uzbekistan	MT526310	[33]
*Laetiporus sulphureus* (Bull.) Murrill	334_YG20211005-1D	*Salix alba* Thunb.	Uzbekistan	OR250352	Present study
*Laetiporus sulphureus*	OLRIM1044	*Quercus robur* L.	Sweden	EU840622	[68]
*Lentinus tigrinus* (Bull.) Fr.	326_YG_MU_1_1	*Platanus orientalis* L.	Uzbekistan	OR250353	Present study
*Lentinus tigrinus*	327_YG_MU_1_2	*Platanus orientalis*	Uzbekistan	OR250355	Present study
*Lentinus tigrinus*	328_YG_N2	*Salix* sp.	Uzbekistan	OR250354	Present study
*Lentinus tigrinus*	336_YG20210725-1D	*Juglans regia*	Uzbekistan	OR250356	Present study
*Lentinus tigrinus*	MG331_K4-2D	*Quercus brantii* Lindl.	Iran	MG208016	[69]
*Sanghuangporus lonicerinus* (Bondartsev) Sheng H. Wu, L.W. Zhou & Y.C. Dai	337_YG20210629-1D	*Lonicera nummulariifolia* Jaub. & Spach	Uzbekistan	OR250357	Present study
*Sanghuangporus lonicerinus*	MG281	Unknown	Iran	KU213574	Unpublished
*Schizophyllum commune* Fr.	YG 047	*Morus alba* L.	Uzbekistan	OR250358	Present study
*Schizophyllum commune*	YG-G42	*Populus* sp.	Uzbekistan	OR250359	Present study
*Schizophyllum commune*	biocode09-540	Unknown	French Polynesia	MZ997086	[70]
*Stereum hirsutum* (Willd.) Pers.	YG-G57	*Juglans regia*	Uzbekistan	OR250361	Present study
*Stereum hirsutum*	YG-G60	*Acer tataricum* subsp. *Semenovii*	Uzbekistan	OR250360	Present study
*Stereum hirsutum*	H21575	*Quercus suber* L.	Tunisia	KU973867	[71]
*Trametes versicolor* (L.) Lloyd	YG-G11	*Crataegus turkestanica*	Uzbekistan	OR250362	Present study
*Trametes versicolor*	YG-G45	*Juglans regia*	Uzbekistan	OR250363	Present study
*Trametes versicolor*	MQN011	Decayed wood	Nepal	AB811857	[72]
*Coniophora arida* (Fr.) P. Karst.	FP-104367	Hardwood	USA	GU187510	[73]
*Gomphidius roseus* (Fr.) Oudem.	MB 95-038	*Pinus sylvestris* L.	Germany	DQ534570	[74]

## 3. Results and Discussion

### 3.1. Species Diversity of Medicinal Basidiomycetes

This study is based on information from the literature and considers morphological and phylogenetic evidence, and we report 17 species of medicinal macrofungi in the study area. Most fresh specimens were collected in the Tashkent Botanical Garden, Jizzakh, Andijan, Tashkent, Fergana, Namangan, and Samarkand provinces of Uzbekistan in 2021–2022. A total of 17 species of medicinal basidiomycetes were identified, belonging to 16 genera (*Auricularia*, *Bjerkandera*, *Cerioporus*, *Cerrena*, *Fomes*, *Fomitopsis*, *Ganoderma*, *Grifola*, *Inonotus*, *Laetiporus*, Lentinus, *Phellinus*, *Sanghuangporus*, *Schizophyllum*, *Stereum*, and *Trametes*) and 11 families (Table 3). In terms of the number of species in the study area, the Polyporaceae family was considered dominant with four species belonging to four genera: *Cerioporus squamosus*, *Fomes fomentarius*, *Lentinus tigrinus*, and *Trametes versicolor*. Macrofungal species of the *Hymenochaetaceae* family belong to three genera: *Inonotus hispidus*, *Phellinus igniarius*, and *Sanghuangporus lonicerinus*. The *Ganodermataceae* family is represented by two species of the *Ganoderma* genus, namely *Ganoderma adspersum* and *G. applanatum*. The families *Auriculariaceae* (*Auricularia mesenterica*), *Cerrenaceae* (*Cerrena unicolor*), *Fomitopsidaceae* (*Fomitopsis betulina*), *Grifolaceae* (*Grifola frondosa*), *Laetiporaceae* (*Laetiporus sulphureus*), *Phanerochaetaceae* (*Bjerkandera adusta*), *Schizophyllaceae* (*Schizophyllum commune*), and *Stereaceae* (*Stereum hirsutum*) are each represented by a single species (Table 3). All these macrofungal species are well-known for their multiple medicinal properties [6,8,9,11].

### 3.2. Phylogenetic Placement of Collections of Medicinal Macrofungi from Uzbekistan

**Figure 2:**
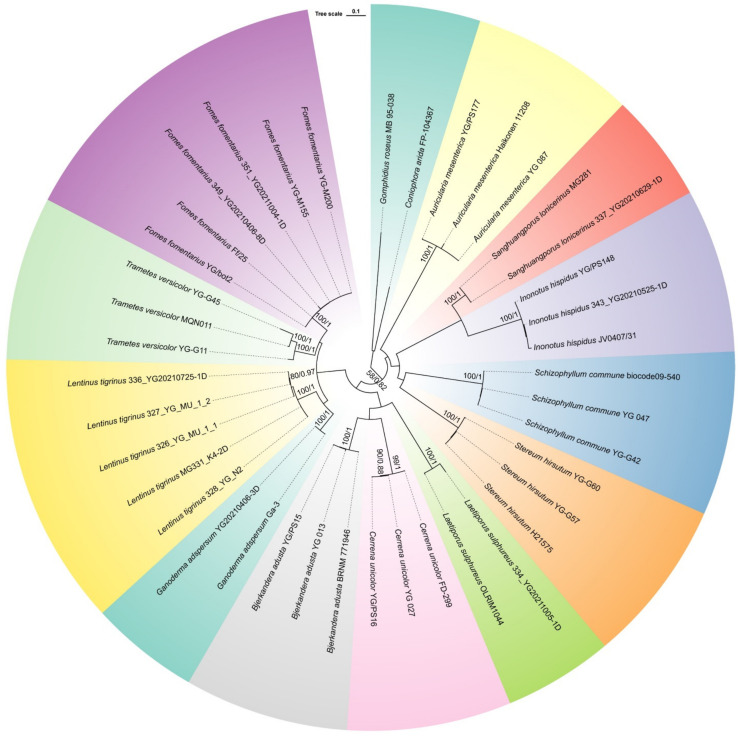
Phylogenetic position of newly sequenced Uzbekistan specimens of medicinal macrofungi inferred from the ITS region. The topology is generated by the maximum likelihood algorithm. The maximum likelihood bootstrap values and the Bayesian posterior probabilities, when simultaneously above 50% and 0.8, respectively, are shown at the nodes. *Coniophora arida* and *Gomphidius roseus* from *Boletales* were selected as outgroup taxa.

In this study, 24 ITS sequences were newly generated from 24 specimens (Table 2). The dataset of the ITS region generated an alignment of 930 characters with GTR + I + G as the best-fit evolutionary model. In the ML algorithm, the BS search stopped after 200 replicates. In the BI algorithm, after 15 million generations with an average standard deviation of split frequencies of 0.002282, all chains converged, which was indicated by the effective sample sizes of all parameters above 8000 and all potential scale reduction factors close to 1.000. Because ML and BI algorithms generated nearly congruent topologies, the topology from the ML algorithm is presented along with BS values and BPPs simultaneously greater than 50% and 0.8, respectively, at the nodes (Figure 2). All newly sequenced specimens grouped together with their corresponding species are represented by the sequences downloaded from GenBank with strong statistical support. This phylogeny confirmed our species morphological identification.

### 3.3. Substrate/Host Preferences of Medicinal Basidiomycetes in Study Area

Medicinal basidiomycetes were found on 23 species of trees and shrubs belonging to 11 families (*Betulaceae*, *Cannabaceae*, *Caprifoliaceae*, *Cupressaceae*, *Fagaceae*, *Juglandaceae*, *Moraceae*, *Platanaceae*, *Rosaceae*, *Salicaceae*, and *Sapindaceae*) and 14 genera (Table 3). The highest number of medicinal basidiomycetes species was reported in the following host genera: Juglans (7 species; 41.17% of the total species number), Acer (5; 29.41%), *Populus* and *Salix* (each 4; 23.52%), *Betula*, *Crataegus*, and *Prunus* (each 2; 11.76%). *Celtis*, *Juniperus*, *Lonicera*, *Quercus*, *Malus*, *Morus*, and *Platanus* plant genera hosts presented a single macrofungal species. Numerous medicinal basidiomycetes were found on walnut (*Juglans regia*), maple (*Acer tataricum* subsp. *Semenovii*), willow (*Salix* spp.), poplar (*Populus* spp.), and other trees. In particular, the cosmopolitan species *Fomes fomentarius*, *Inonotus hispidus*, *Lentinus tigrinus*, *Schizophyllum commune*, and *Stereum hirsutum* are widespread and harmful to plants belonging to the families *Cannabaceae*, *Juglandaceae*, *Moraceae*, *Platanaceae*, *Rosaceae*, *Salicaceae*, and *Sapindaceae*. The previously cited macrofungi are multi-hosted on trees and shrubs belonging to different plant families. The reason for the regularity of this distribution of macrofungi may be related to the predominance of hardwoods in the flora of Uzbekistan [32,33,37]; these macrofungal species have caused various diseases and decay of the woody plants [36,75,76]. Currently, 153 wood-inhabiting poroid and corticioid basidiomycetes have been recorded on more than 100 species of woody plants in Uzbekistan [33,36,37].

### 3.4. Distribution of Medicinal Basidiomycetes by Areas of Uzbekistan

**Figure 3:**
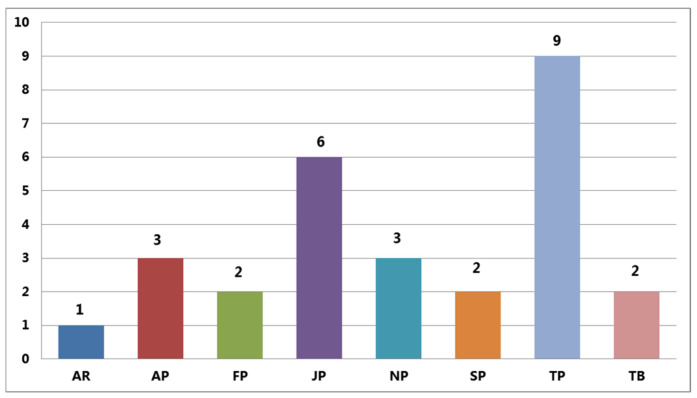
Distribution of medicinal macrofungi in provinces of Uzbekistan. The names of the mushroom distribution areas are abbreviated as follows: All region—AR; Jizzakh province—JP; Tashkent province—TP; Namangan province—NP; Fergana province—FP; Andijan province—AP; Samarkand province—SP; Tashkent Botanical Garden—TB.

An analysis of the distribution of medicinal basidiomycetes, most often collected in urban and mountainous areas of Tashkent province, revealed nine species (*Auricularia mesenterica*, *Fomitopsis betulina*, *Fomes fomentarius*, *Lentinus tigrinus*, *Cerrena unicolor*, *Trametes versicolor*, *Phellinus igniarius*, *Sanghuangporus lonicerinus*, and *Stereum hirsutum*), which constitute 52.95% of the total medicinal basidiomycete studies in this study area. The most abundant macrofungi were found in Jizzakh province, with six species records mainly distributed in Juniper forests of the Turkestan mountain range in the Zaamin district of Jizzakh (*Bjerkandera adusta*, *Fomitopsis betulina*, *Laetiporus sulphureus*, *Phellinus igniarius*, *Sanghuangporus lonicerinus*, and *Stereum hirsutum*), which constitute 35.29% of the total number of medicinal macrofungi. In the provinces of Namangan and Andijan, three species were found in each province, representing 17.64% of the total mycobiota. Macrofungal species were rare in the urban and mountain forests in Fergana, Samarkand provinces, and the Tashkent Botanical Garden where only *Inonotus hispidus* and *Stereum hirsutum* were found. Figure 3 shows the distribution of basidiomycetes in the study areas.

### 3.5. Taxonomic Treatment, Distribution, Ecology of Medicinal Macrofungi, and Their Uses in Ethnomedicine, Modern Medicine, and Other Fields

Throughout human history, people have maintained a deep connection with macrofungi, drawing on their folk knowledge for medicinal and culinary purposes. Among these, the traditional expertise on medicinal mushrooms holds a significant and irreplaceable role in human life. It is well-documented that ancient Chinese and Oriental folk medicine extensively utilized various fungi to effectively treat a wide range of human diseases. Avicenna, namely Abu Ali Hussin bin Abdallah ibn Hasan ibn Ali ibn Sino was born in 980 in the village of Afshana, the present-day Bukhara region in Uzbekistan; he described truffles as a cure for a variety of disorders, i.e., vomiting, wounds, and weakness in the “*Al-Qanun fi’t-Tibb*” (*The Canon of Medicine*) [77] written in the 10th century as part of the Arabian Traditional Medicine. Avicenna recommended desert truffle juice used for common eye inflammations [78]. *The Canon of Medicine* was used as a medical textbook in the Islamic world and Europe up to the 18th century. At the same time, Al-Biruni, namely Abu Raihon Muhammad ibn Ahmad Al Beruniy, born in Khorazm in Kiyat city in 973 in Uzbekistan; in his book “*Kitab as Saidana fi-t-tibb*” [79], morel (*Morchella esculenta*) is mentioned as a laundry detergent. When dried, the morels become white and inside, red. They are put in an antimony vessel andhave the color of dust with a black tint, and are a medicine for the eyes, applying such an ointment with a stick [78]. However, despite these steps forward, comprehensive information on the edible and medicinal macrofungi is still unavailable in Uzbekistan in many aspects. In this article, we present current ethnomycological and medicinal uses, as well as other uses of medicinal macrofungi in Uzbekistan based on the literature review and our own data.

#### 3.5.1. *Auricularia mesenterica* (Dicks.) Pers., Mycologia Europaea 1: 97 (1822) (Table 3; Figure 4A)

Ethnomedicinal uses: In the past, *A. mesenterica* (Tripe Fungus) was used in Europe to treat throat ailments after being boiled in beer, milk, or vinegar, and it was often used to treat eye ailments [80]. In Asian countries, *A. mesenterica* has been traditionally used as natural medicine; the fruiting bodies were used in China for cold and fever treatments, in addition to being employed to cure a variety of ailments, such as hemorrhoids, hemoptysis, and angina, as well as to strengthen the body [80]. In Mexico, *A. mesenterica* was utilized in traditional medicine against anxiety and fear [81].

**Figure 4:**
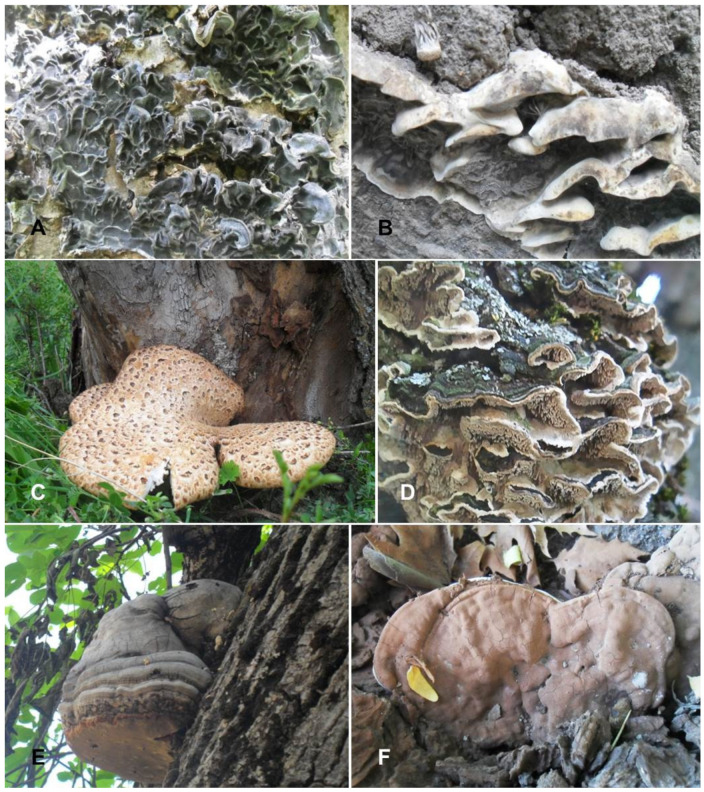
*Auricularia mesenterica* (**A**), *Bjerkandera adusta* (**B**), *Cerioporus squamosus* (**C**), *Cerrena unicolor* (**D**), *Fomes fomentarius* (**E**), and *Ganoderma adspersum* (**F**). Photo credit by Yusufjon Gafforov.

Medicinal uses: *Auricularia mesenterica*, like other species of the genus *Auricularia*, has several pharmacological benefits including antioxidant and anti-inflammatory effects [82,83,84]. Antioxidant properties were tested by Payamnoor et al. [85] and resulted in both methanol and ethanol extracts having high antioxidant properties; the results of this study suggested that *A. mesenterica* should be a good source for the designing of different drugs. Indeed, the production of antioxidant compounds, phenolic acids, flavonoids, and ascorbic acid, gives *A. mesenterica* antioxidant properties and these compounds act by inhibiting free radical chain formation and chelating transition metal ions, as well as scavenging reactive oxygen species [86].

Morphological descriptions: Basidiomata can be solitary or caespitose, rubbery, gelatinous, and resupinate to effused-reflexed. Pileus is free-lobed, 3.6–7.2 × 2.6–5.6 cm in diameter, 3.5 mm thick when fresh, and 0.2–0.3 mm thick when dry. The upper surface is hispid, distinctly and concentrically zoned with canescent zones and dark bands, becoming olivaceous buff upon drying, smooth, and hairy. Hymenophore porose˗reticulate, vined-wrinkled, surface venose with obvious folds, greyish ruby, becoming fawn to reddish brown upon drying, clamp connection present in all tissues. Medulla and crystals absent; abhymenial hairs irregular, with a slightly swollen base, hyaline, thick-walled with a narrow lumen, apical tips acute or obtuse, tufted, 1000–2000 × 2–4 µm. Hyphae hyaline, without clamp connections, 2–12 µm in diameter. Basidia cylindrical-conic, clavate, transversely 3-septate, 34.48–40.94 × 4.31–6.46 μm, sterigmata prominent, 4.31–15.08 × 1.29–2.15 μm. Cystidioles present, 5–9 × 2.5–5 µm. Basidiospores cylindric, allantoid, hyaline, thin-walled, smooth, usually with one or two large guttules, (13.8–)14–17(–17.6) × (4.5–)4.7–5.2(–5.3) µm [86,87].

Distribution and habitat: *A. mesenterica* was first described in Northern and Central Europe; it grows on different woody angiosperm plants [87]. *A. mesenterica* is also reported in North and South America [88,89] as well as Africa, Australia, Asia [90], and Uzbekistan [39]. In Uzbekistan, *A. mesenterica* commonly grows on angiosperm wood plant species that are usually found throughout the year.

Edibility, aroma, and flavor: Edible, mild odor, flavor, tough and leathery.

#### 3.5.2. *Bjerkandera adusta* (Willd.) P. Karst., Meddelanden af Societas pro Fauna et Flora Fennica 5: 38 (1879) (Table 3; Figure 4B)

Ethnomedicinal uses: It was used in traditional Chinese medicine (TCM) for the treatment of uterine cancer [91,92].

Medicinal uses: *B. adusta* commonly known as the smoky polypore or smoky bracket was investigated for various pharmacological properties as its antibacterial and antifungal properties. It is widely used in many countries in the treatment of inflammation in the human body and in diseases associated with various microorganisms, fungi, and viruses [93,94,95]. It is important to mention that *B. adusta* is one of the most significant etiological fungi associated with chronic cough [96,97,98].

Morphological descriptions: Basidiomata annual, sessile, pileate to effused-reflexed, rarely resupinate, 5–7 cm long and 2–5 cm wide. Upper surface tomentose, strigose, azonate or faintly zonate, later glabrous, cream to brown, ochraceous or greyish. Margin wavy, whitish-greyish in color. *Hymenophore* grey to black pores round-angular, 5–7 per mm, dissepiments thin and entire. Context whitish to grey or pale ochraceous, azonate, up to 10 mm thick, separated by a thin grey to black layer from the upper part of the tube layer. Tube layer grey to smoky up to 1–2 mm thick. Hyphal system monomitic, generative hyphae colorless, thin- or thick-walled, branched, with clamps, 3–5 µm wide, agglutinated, and compact in the trama. Cystidia and cystidioles were absent. Basidia are clavate, have 4 sterigmata, and are 10–15 × 3.5–5.5 μm. Basidiospores are subcylindrical, colorless, smooth, and are 4.3–6 × (2.4–)2.6–3.1(–3.5) μm [99].

Distribution and habitat: It mostly occurs on various dead deciduous trees, rarely on coniferous trees. The species is widespread through temperate and boreal Eurasia, reported also from the tropics and temperate southern hemisphere [100,101,102]. It is mostly found on *Juglans*, *Populus*, and *Prunus* species in Uzbekistan [33].

Edibility, aroma, and flavor: Inedible, smells intense and pleasant when fresh.

**Table 3:** Ethnomycology and ethnomedicinal uses of the wood-inhabiting basidiomycete macrofungi from Uzbekistan (species name, local name, folk and modern medicinal uses, host plants).

Species/Family	Local Name	Used Method	Host Plants	Medicinal Uses		References
				Ethno Medicine	Modern Medicine	
*Auricularia mesenterica Auriculariaceae*	Jigarrang quloq qo‘ziqorini	Edible, medicinal	*Prunus vulgaris*, *Prunus armeniaca*	Treats throat, cold, and fever, hemorrhoids, haemoptysis, and angina. Used against anxiety and fear	Anti-inflammatory, antioxidant	[37,80,81,82,83,84,85,86]
*Bjerkandera adusta* *Phanerochaetaceae*	Tutinsimon pukak	medicinal	*Juglans regia*, *Juniperus* sp., *Populus alba*	Treat uterine cancer	Antibacterial, antifungal, antioxidant	[37,91,92,93,94,95,96,97]
*Cerioporus squamosus* *Polyporaceae*	Tangachali po‘kak, tangachasimon polipor	Edible, medicinal	*Juglans regia*	Pain reliever and in the treatment of joint diseases, tuberculosis, pneumonia, bronchitis, dysfunction of the kidneys and bladder; oncology; gout; problems with the functioning of the pancreas; liver pathology; ulcer and gastritis	Antibacterial, anticancer, antifungal, antioxidant, immunomodulating, immunosuppressive	[37,103,104,105,106,107,108,109,110,111]
*Cerrena unicolor* *Cerrenaceae*	Tserrena birxil rangli, moxli labirent po‘kak	Medicinal	*Acer tataricum* subsp. semenovii, *Crataegus* *turkestanica*	Treatment of many human diseases. Tincture also used as an antiseptic	Anticancer, antidiabetic, antifungal, antimelanomic, antimicrobial, antiparasitic, antioxidant	[37,112,113,114,115,116,117,118,119,120,121,122]
*Fomes fomentarius* *Polyporaceae*	Haqiqiy buqoq zamburug‘i, Xaqiqiy pukak	Medicinal	*Betula tianschanica*, *Juglans regia*, *Malus domestica*	Bladder disorders, dysmenorrhoea, hemorrhoids, gastrointestinal disorders, hepatocirrhosis, oral ulcers, and inflammation. Esophagus, gastric, and uterus carcinoma	Anticancer, antibacterial, antidiabetic, anti-inflammatory, antimicrobial, antiseptic, antiviral, antioxidant	[37,123,124,125,126,127,128,129,130,131,132,133,134,135]
*Fomitopsis betulina* *Fomitopsidaceae*	Oqqayin pukak zamburug‘, oqqayin fomitopsis	Medicinal	*Betula tianschanica*	Used as tinder and anesthetic, anti-fatiguing, soothing, and for immunoenhancing properties. Treatment of rectal cancer and stomach diseases; also used against various cancer types, as an immunoenhancing. Antiparasitic agent, and a remedy for gastrointestinal disorders	Antibacterial, anti-inflammatory, antimicrobial, antioxidant, antiseptic, antiviral, cytotoxic	[37,136,137,138,139,140,141,142,143]
*Ganoderma applanatum* *Ganodermataceae*	Tug‘ri pukak, Ayiq noni	Medicinal	*Populus* sp., *Salix* sp.	Reduces excess phlegm, pain, and fever; lowers blood glucose and improves immunity, hypertension, asthma, bronchitis, prostatitis, and mental disorders	Antiallergic, anticancer, antifibrotic, antihyperglycemic, anti-inflammatory, antimicrobial, antioxidant, antitumor, hepatoprotective, hypoglycemic, immunomodulatory	[8,9,37,124,126,130,144,145,146,147,148]
*Ganoderma adspersum* *Ganodermataceae*	Pog‘anasimon ganoderma xilma-xil po‘kak	Medicinal	*Acer saccharum*	Treats neurasthenia, nervous diseases, hypertension, liver and cardiovascular diseases, diseases of the genitourinary system, diabetes, rheumatism, gastritis, ulcers, cancer and tumors	Antiatherosclerotic, antifungal, anti-inflammatory, antioxidant, antitumor, antiviral, antidiabetic, hepatoprotective, and neuroprotective	[37,66,130,148,149,150,151,152,153,154,155]
*Grifola frondosa* *Grifolaceae*	Jingalak grifola zamburug‘, qo‘chqorbosh zamburug‘	Edible, medicinal	*Quercus* sp.	Treats cancer, diabetes, and inflammation.	Anti-aging, antiallergic, antidepressant, antidiabetic, anti-inflammatory, antimicrobial, antioxidant, antitumor, antiviral, hepatoprotective, hypoglycemic, hypolipidemic, immunomodulatory, immunostimulatory, neuroprotective, nephroprotective	[37,156,157,158,159,160,161,162,163,164,165,166]
*Inonotus hispidus* *Hymenochaetaceae*	Yolli inonotus	Inedible, medicinal	*Populus pruinosa* Schrenk, *Juglans regia*, *Morus alba*	Used as anthelminthic, against diarrhea and for general internal cleaning. Treats heart, liver disease, stomach diseases and stomach-ache Used to wash external the sexual organs during menstruation and after birth to clean the body	Antifungal, anti-inflammatory, antioxidant, antimicrobial, antiproliferative, antitumor, antiviral, immunomodulatory, immunostimulatory, hypolipidemic	[37,167,168,169,170,171,172,173]
*Laetiporus sulphureus* *Laetiporaceae*	Kulrang-sarg‘ish po‘kak, Oltinrang po‘kak	Medicinal	*Acer tataricum* subsp. *semenovii*, *Salix alba*	Widely used for treatment of pyretic diseases, coughs, gastric cancer, and rheumatism. Burning fruit bodies is presumed to drive away mosquitoes and midges	Anticoagulant, anti-inflammatory, antimicrobial, antitumor, antioxidant, cytostatic, hypoglycemic and immunostimulative	[17,37,111,114,124,174,175]
*Lentinus tigrinus* *Polyporaceae*	Chuqurchasimon pukak, bahorgi pukak zamburug‘	Edible, medicinal	*Salix* sp., *Platanus orientalis*, *Juglans regia*	Used as food, medicine, brain tonic, and against anger. Powder taken in hot water relieves dry cough and asthma, arthritis, colds, fever, headache, hypertension, skin diseases, stomach-ache, and toothache	Anticancer, antidiabetic, anti-inflammatory, antimicrobial, antioxidant, immunomodulatory	[37,124,176,177,178,179,180,181,182,183]
*Phellinus igniarius* *Hymenochaetaceae*	Majnuntol pukak	Inedible, medicinal	*Salix* sp., *Acer* sp.	Used when endometrorrhagia, heart attack, or stroke, to reduce the risk of cancer, and decrease various inflammations	Antiangiogenic, anticancer, anti-inflammatory, antioxidant, antiviral, neuroprotective	[37,66,109,148,184,185,186,187,188,189]
*Sanghuangporus lonicerinus* *Hymenochaetaceae*	Uchqat sangxuangporusi	Inedible, medicinal	*Lonicera* sp., *Lonicera nummulariifolia*	–	Antiproliferative, cytotoxic, estrogenic and anti-estrogenic, hepatoprotective	[37,185,186,190,191]
*Schizophyllum commune* *Schizophyllaceae*	Oddiy jabrasimon zamburug‘	Inedible, medicinal	*Morus alba*, *Juglans regia*, *Celtis caucasica* Willd., *Populus* sp.	Regulates blood pressure. Treats leucorrhea and breast inflammation	Analgesic, anti-constipation, antidiabetic, anti-inflammatory, antimicrobial, antioxidant, antitumor, chemoprotective, cosmeceutical agent, immunostimulating, neuroprotective, and wound healing properties	[37,111,192,193,194,195,196,197,198,199,200,201,202]
*Stereum hirsutum Stereaceae*	Soxta kurka dumi zamburug‘i, tukli pardali kurka	Inedible, medicinal	*Juglans regia*, *Acer tataricum* subsp. *semenovii*	Used to treat cancer, diabetes, and dyspepsia, and as antiseptic	Antidiabetic, anti-inflammatory, antimicrobial, antioxidant, cytotoxic, anti-lipase, neuroprotective	[37,126,203,204,205,206,207,208]
*Trametes versicolor* *Polyporaceae*	Turli-xil rangli pukak	Inedible, medicinal	*Prunus* sp., *Juglans regia*, *Crataegus turkestanica*	Used in liver disorders such as hepatitis, jaundice, and liver cancer as well as heart, gastric, and kidney disorders. Used in asthma, cough, fever, herpes, and in pathologies of the upper respiratory tract, urinary and digestive infections. Boosts immune functions, Treats chronic fatigue syndrome, Promotes increased energy	Anti-acetylcholinesterase, anticancer, antidiabetic, anticoagulant, anti-inflammatory, antimicrobial, anti-obesity, antioxidant, antitumor, cardioprotective, hypocholesterolemic, hypolipidemic, hepatoprotective, immunoregulatory, immunostimulatory	[37,124,208,209,210,211,212,213]

#### 3.5.3. *Cerioporus squamosus* (Huds.) Quél., Enchiridion Fungorum in Europa Media et Praesertim in Gallia Vigentium: 167 (1886) (Table 3; Figure 4C)

Ethnomedicinal uses: In TCM, this fungus was used as a pain reliever for the treatment of joint diseases [109]. The list of diseases for which drugs based on the scaly polypore named Dryad’s saddle are used is quite extensive: diseases of the bronchi and lungs (tuberculosis, pneumonia, bronchitis); dysfunction of the kidneys and bladder; oncology; gout; problems with the functioning of the pancreas; liver pathology; ulcer and gastritis; obesity. It is used to recover from injuries.

Medicinal uses: This fungus is a basidiomycete bracket fungus widely used in medicine due to the presence of various biologically active substances as antibacterial [108], anticancer [111], antifungal [110], antioxidant [105,106,108], diuretic [107], immunomodulating and immunosuppressive [103,104] agents.

Morphological descriptions: Basidiomes annual, laterally stipitate, large up to 40–60 cm wide and up to 6–8 cm thick, kidney-shaped to nearly circular. Upper surface cream, ochraceous with a thin blackish-brown pellicle that breaks off to form large, concentrically arranged dark brown scales, becoming clumped with age. Margin undulating, concolorous with the upper surface. The pore surface descends along the stem; whitish to cream, becoming yellowish to pale brown with age. Pores angular, 1–2 per mm wide, context corky and azonate, brittle when dry, cream to ochraceous, up to 4 cm thick. Tube layer decurrent to the stipe, concolorous with the context, up to 1.5 cm thick. Stem 3–12 × 1–6 cm, short, sometimes rudimentary usually lateral, hard, blackish to black. Hyphal system dimitic: generative hyphae thin-walled, clamped, colorless, 2–5 µm in diameter. Skeleto-binding hyphae in context thick-walled, sometimes branched, colorless 4–7.5 µm wide, tramal hyphae similar, monomitic in young basidiomes. Hymenial cystidia absent, cystidioles present, fusoid, 20–35 × 5– µm. Basidia clavate, with 4 sterigmata, 40–80 × 9–12 µm. Basidiospores are broadly ellipsoid to cylindric, smooth, colorless, 10–17 × 4.5–6 µm [99].

Distribution and habitat: It occurs in temperate and boreal zones of the northern hemisphere. Ryvarden and Gilbertson [214] reported 26 host genera including one conifer (*Larix*). It was found on the dried trunk of *Acer tataricum* subsp. *semenovii*, *Juglans regia*, *Pistacia* sp., *Populus alba* and *Quercus* sp. in Uzbekistan [33,102].

Edibility, aroma, and flavor: Fragrant, strong flavor, flour or mushroom odor, persisting long in later dried specimens; fruit body becomes hard and rough. It seems edible when young.

#### 3.5.4. *Cerrena unicolor* (Bull.) Murrill, J. Mycol. 9 (2): 91 (1903) (Table 3; Figure 4D)

Ethnomedicinal uses: *C. unicolor* has long been used as a traditional Chinese medicinal mushroom. It was widely used in Asian countries for the treatment of many human diseases and its tincture was applied as an antiseptic [112,114].

Medicinal uses: *C. unicolor*, commonly known as the mossy maze polypore, is still being studied scientifically in traditional medicine. According to modern scientific data, *C. unicolor* synthesizes secondary metabolites with a broad spectrum of biological activities [117,120]. In addition to antidiabetic properties, antimicrobial, and hepatoprotective activities of exopolysaccharides of *C. unicolor* were also investigated in recent years [114,115,116]. The results obtained so far have also shown antiproliferative [118,121], proapoptotic, and migration-inhibiting [121] properties of low molecular weight sub-fractions from the *C. unicolor* secretome, especially towards HT-29 colon cancer cells [119,121,122].

Morphological descriptions: Basidiomata annual, imbricate, laterally fused, effused-reflexed to rarely resupinate, and up to 10 cm wide and roughly 5 mm thick. Surface concentrically zonate, hirsute, covered in tufts of hyphae, to almost glabrous, grey to brown, and frequently green from algae. Margin thin and wavy. Hymenophore is creamy, ivory, ages to grey, then turns brown. Pores irregular, daedaleoid to labyrinthine, 2–3(4) per mm; dissepiments thick and tomentose. Context duplex, up to 3 mm thick, lower layer pale brownish and corky, upper part soft, spongy, and darker separated by a thin dark zone. The tube layer is concolorous with the lower layer of context, up to 1 cm thick. Cystidia absent, long terminal clavate hyphal ends with thickened walls present on dissepiment edges and in old hymenium, projecting 4–6 µm and are 2–3 µm wide; fusoid cystidioles in hymenium thin-walled 16–20 × 4–6 µm. Hyphal system trimitic: generative hyphae thin-walled, clamped, 2–4 µm wide; skeletal hyphae non-septate, thick-walled, rarely branched, 2.5–5 µm wide; binding hyphae non-septate, thick-walled, abundantly branched, 2–4 µm wide. Basidia clavate with a basal clamp and 4 sterigmata, 18–25 × 5–6 µm. Basidiospores are cylindric to ellipsoid, colorless, smooth, 5–7 × 2.5–4 µm [99].

Distribution and habitat: Widely distributed in Asia, Europe, and North America from the Mediterranean to the boreal zone [215]. It grows on dead or living, weakened broadleaved trees. It occurs on the trees of *Celtis*, *Crataegus*, *Juglans*, *Populus*, and *Quercus* in Uzbekistan [33].

Edibility, aroma, and flavor: Not distinctive, inedible.

#### 3.5.5. *Fomes fomentarius* (L.) Fr., Summa vegetabilium Scandinaviae 2: 237 (1849) (Table 3; Figure 4E)

Ethnomedicinal uses: As early as 400 BCE, Hippocrates referring to *F. fomentarius* used the Greek term “mykes”; this suggests that *F. fomentarius* commonly known as the tinder fungus is one of the earliest mushrooms to have been cited [216]. Native Americans used the fungus as a diuretic and laxative, calming the nerves and against rheumatism [109,138]. *F. fomentarius* was available in pharmacies in Europe until the 19th century under the name *Fungus chirurgorum* (wound sponge); it was applied by surgeons, dentists (during dental extractions), and barbers as a hemostatic wound dressing as well as warming compresses and cautery for moxibustion. The fungus extract was used to treat bladder issues, dysmenorrhea, and hemorrhoids to relieve pain [123,141,216,217]. In traditional Chinese and Korean medicine, *F. fomentarius* was administered orally to treat gastrointestinal disorders, cirrhosis, oral ulcers, and inflammation. Furthermore, it is still being recommended as a supportive medicine for esophagus, gastric, and uterus carcinoma [127,138].

Medicinal uses: For ages, *F. fomentarius* helps to heal wounds. Clinical trials on patients with wounds, burns, and trophic ulcers indicated that topical application of the fibers resulted in stopping the bleeding, reducing pain, and preventing inflammation and suppuration [123]. Furthermore, *F. fomentarius* has diverse medicinal applications including anti-cancer [125,133], anti-diabetes [129], antimicrobial [128,131], anti-inflammatory [126], and antioxidant activities [132,134,135].

Local handicraft and other uses: The first evidence of using the fluffy, felted middle layer (amadou) of *F. fomentarius* dates back to about 5300 years ago. The species was identified in a first aid kit of the world-famous glacier mummy “Ötzi” (named after Ötztal, the valley on the borders between Austria and Italy where he was discovered); it was used as a tinder material and for spiritual purposes. Peintner and Pöder [218] pointed out the multiple uses of this fungus: fire production, styptic dressing, medicine, clothes, fishing articles, and decorative items as well as its symbolic and legendary role. The mentioned experiments on fire production and clothing fabrication demonstrate the efficiency of traditional techniques [123]. *F. fomentarius* fruiting bodies were also used as snuff or for ritual smoking ceremonies in Eastern America, Austria, France, Germany, Japan, and Western Siberia [109,138,141,219].

Morphological descriptions: Basidiomata pileate, perennial, sessile, woody hard, up to 40 cm wide. The upper surface is smooth, pale brown, reddish brown to grey. The margin is whitish to pale brown. Hymenophore poroid, pore surface ochraceous to grey, darker when touched or incised, 27–30 pores/cm thick. Context brownish, 2–3 cm thick, tough-fibrous, homogenous, black with KOH. Hyphal system trimitic: generative hyphae thin-walled, colorless, branched, with clamps 2–4 µm wide, inconspicuous; skeletal hyphae thick-walled, aseptate, pale yellowish brown, brown with KOH, 3–8 µm wide; binding hyphae yellowish brown, thick-walled, much branched, aseptate, 1.5–3 µm in diameter. Cystidia absent, cystidioles common in the hymenium, thin-walled, fusoid, 24–40 × 3.5–7 µm with a basal clamp emerging from hymenial layer; also, hyphoid apical tips of encrusted hyphae appear at dissepiment edges. Basidia are cylindric, with 4 sterigmata, and 21–25 × 7–9 µm. Basidiospores are cylindric, thin-walled, smooth, and (10)12–22 × (4.4)5–8(10) µm [99].

Distribution and habitat: *F. fomentarius* grows on living and dead trees of hardwoods, and rarely on conifers [220]. *F. fomentarius* has been reported on *Juglans*, *Malus*, *Populus*, *Quercus*, and *Salix* trees in Uzbekistan [33,102].

Edibility, aroma, and flavor: Inedible species with a fruity smell. It is acrid in taste.

#### 3.5.6. *Fomitopsis betulina* (Bull.) B.K. Cui, M.L. Han and Y.C. Dai., Fungal Diversity 80: 359 (2016) (Table 3)

Ethnomedicinal uses: This species (previously named *Piptoporus betulinus*) is commonly known as the birch polypore; it is one of the most common species in central Europe and has been commonly used in folk medicine, especially in Baltic countries [140]. It has also been used as tinder and anesthetic [140]. In Russia, tea of the fungus was thought to have anti-fatiguing, soothing, and immunoenhancing properties. *F. betulina* was used in Bohemia for the treatment of rectal cancer and stomach diseases [141]. Interestingly, antiseptic and pain reliever applications of the polypore were reported in Europe and the USA [138,141]. *F. betulina* has been traditionally exploited as an antiparasitic, antimicrobial agent in the treatment of wounds for staunching bleeding [136]. Infusion from *F. betulina* basidiome was popular, especially in Russia and Baltic countries, Hungary, and Romania for its nutritional and calming properties. Fungal tea was used against various cancer types, as an immunoenhancing, antiparasitic agent, and a remedy for gastrointestinal disorders [137,141,142]. Antiseptic and anti-bleeding dressings made from fresh *F. betulina* fruiting body were applied to wounds and the powder obtained from dried ones was used as a painkiller [139,142,221].

Medicinal uses: Due to the broad spectrum of its phytochemical composition, this species has amazing diverse pharmacological properties such as anti-inflammatory, antiseptic, and antibacterial benefits. In addition, the astringent effect alone makes it good for making mushroom-containing herb tea or an immune tonic to be taken once a week to boost the immune system. *F. betulina* contains primary metabolites (as polysaccharides) and secondary metabolites (as triterpenes) that are beneficial for health [6,143,222].

Other uses: The velvety cut surface of the fruit body was traditionally used as a strop for finishing the edges of razors [223]. Additionally, it was apparently also described for fine metal polishing, making ink blotters, and mounting insect collections. One use that would have been important in ancient times is that *F. betulina* takes a spark well and can be used to carry fire over long distances. Therefore, it allowed people to move around while maintaining easy access to fire [223].

Morphological descriptions: Basidiomata perennial, annual, rounded to substipitate, fleshy, leathery and elastic, 3–15 cm overhanging, 4.5–12.5 cm wide, 1.7–3.5 cm thick towards base, sessile, dimidiate, reniform to suborbicular, often pendent, often convex to flat, pulvinate, velvety at first and covered with a fine smooth cuticle, which cracks and exfoliates to give a pitted to scaly appearance with age, white, beige to pale brown, with a straight to slightly wavy margin. Margin often curved and formed a large bead folded down on the poroid side, concolorous, white to cream at first, and pale brownish with age. Hymenium white to ochraceous when old, pores round to angular, 3–5 per mm, with thick and entire dissepiments at first, splitting, lacerating, and becoming hydnoid with age. Tube layer easily detachable from context, up to 10 mm thick. Stipe up to 7 × 5 cm, short, rudimentary, lateral to slightly overhanging the cap, glabrous, whitish to brown. Context homogenous, soft when dry, white to creamy, tough when dry, up to 5 cm thick. Hyphal system di-trimitic: generative hyphae thin-walled, branched, colorless, 2–5 µm wide; skeletal hyphae from context thick-walled to solid, aseptate, rarely branched, colorless, 2–6 µm wide.; some hyphae are thick-walled but often branched, with broad lumen, up to 11 µm wide. Cystidia and other sterile hymenial elements are absent. Basidia clavate, with 4 sterigmata, clamped at the base, 12–15 × 4–6 µm. Basidiospores are cylindrical to suballantoid, smooth, colorless, 4.5–6 × 1.5–1.7 µm [99].

Distribution and habitat: The geographic distribution of *F. betulina* is restricted to the northern hemisphere, including Europe, Asia, North Africa, and North America [99,224]. This species was recorded on *Betula tianschanica* and unidentified birches (*Betula* sp.) from Uzbekistan [33].

Edibility, aroma, and flavor: Slightly bitter, strong and pleasant odor and an astringent bitter taste, inedible.

#### 3.5.7. *Ganoderma adspersum* (Schulzer) Donk, Proceedings van de Koninklijke Nederlandse Akademie van Wetenschappen Section C 72: 273 (1969) (Table 3; Figure 4F)

Ethnomedicinal uses: Fruiting bodies of *G. adspersum* have been studied at the Neolithic site of La Draga (Spain), confirming the ethnomycological importance of the species in Europe in prehistoric times.

Medicinal uses: The bioactive metabolites of *Ganoderma* species are reported to be responsible for antiatherosclerotic, antidiabetic, anti-inflammatory, antimicrobial, antioxidant, antitumor, hepatoprotective, and neuroprotective activities [150,151,154]. Ganoderma species are also used as functional food to prevent and treat a lot of diseases including anorexia, arthritis, asthma, cardiovascular problems, diabetes, gastritis, hepatitis, hypercholesterolemia, hypertension, insomnia, migraine, nephritis, obesity, and tumorigenesis, amongst others [154]. *Ganoderma adspersum* has medicinal properties, such as antidiabetic [148], antimicrobial [66,155], antioxidant [148,152,153], antitumor [149], and neuroprotective [130,148,152,153] activities as well as anti-tyrosinase activity reported in the mycelia and fruiting bodies of *G. adspersum*.

Morphological descriptions: Basidiomata perennial, flat to hoofed, solitary to imbricate, 5–75 cm long and wide, up to 20 cm thick, dull surface, leathery, chocolate brown/rusty/chestnut to gray-brown, uneven with zones of concentric circles, crust hard brown; Margin white with indistinct yellow zone to slightly pale brown or reddish brown; Hymenophore whitish to ochraceous and brown, darker when touched. *Pores* round 3–4 per mm, thick. Tube layer dark reddish brown, stratified, 6–8 cm thick. *Context* homogenous, brown to reddish brown, 10–15 cm thick. Hyphal system trimitic: generative hyphae with clamps 1.5–3.5 µm, colorless, branched, thin-walled; vegetative hyphae 4–5 (–6) µm wide, abundantly branched at the top, yellow-brown, aseptate, tapering ends; binding hyphae few. Cystidia is absent. Basidia are 22–30 × 7–12 µm, colorless, broadly club-shaped, have 4 sterigmatic with a basal clamp. Basidiospores are (9–) 9.5–11 (–12.7) × (5.5–) 6–7.5 (–8.5) µm ellipsoid, truncated, ornamented, and pale brown [28,99].

Distribution and habitat: Occurs in the temperate region of Eurasia. The fungus has been recorded almost exclusively on hardwoods; it was found on a wide range of living deciduous trees, e.g., *Alnus*, *Fagus*, *Fraxinus*, *Juglans*, *Morus*, *Pinus* sp., *Platanus*, *Robinia*, *Prunus*, and *Quercus*, rarely on conifers trees [28,33,99].

Edibility, aroma, and flavor: Inedible, not significant odor but bitter taste.

#### 3.5.8. *Ganoderma applanatum* (Pers.) Pat., Bulletin de la Société Mycologique de France 5: 67 (1889) (Table 3)

Ethnomedicinal uses: It has been used in traditional medicine for many years in the form of a tea or an aqueous extract, which generally imparts a sensation of intense heat when consumed. The taste and smell of this fungus may vary depending on the host plant [225]. In TCM, *G. applanatum* is used for digestive disorders to reduce excess phlegm, pain, and fever, lowering blood glucose and improving immunity. Additionally, it is used as an antiviral and antitumor agent [225]. Additionally, the health problems associated with potency, hypertension, asthma, bronchitis, prostatitis, mental disorders, and cardiovascular and circulatory systems were also largely solved. Since ancient times, *G. applanatum* has been used in eastern Asia for its medicinal benefits in treating conditions like cancer, hepatitis, diabetes, erectile dysfunction, hypertension, asthma, bronchitis, prostate, and mental disorders as well as for the proper operation of the cardiovascular and circulatory systems (https://progrib.ru/trutoviki/trutovik-ploskiy.html, accessed on 3 June 2023). In Nigeria, *G. applanatum* has been used as an antioxidant, hypoglycemic, and antihypertension agent [226]. It has been used to treat internal growth, heart problems, and cancer in Cameroon [145]. The powder of dried *G. applanatum* fruiting body is added to vegetables in very small quantities during cooking with the belief that it reduces the chances of disease in India [227]. It has also been used in Serbia for strengthening the immune system as tea [18].

Medicinal uses: *G. applanatum* (Artist’s conk) produces various bioactive compounds that exhibit antiallergic, anticancer, antifibrotic, antihyperglycemic, antimicrobial, antioxidant, antitumor, hepatoprotective, hypoglycemic, immunomodulatory, liver protective properties as well as inhibition of aldose reductase enzyme, Epstein–Barr and influenza virus [9,11,124,146,148,154]. Currently, *G. applanatum* and other species of the genus *Ganoderma* are used in China and Japan for the treatment and prevention of hepatitis, hypertension, chronic bronchitis, bronchial asthma, hyperglycemia, rheumatism, connective tissue, and esophageal cancer (a dangerous tumor with epithelial cells), arthritis, tuberculosis, and many other diseases [144,147,228,229].

Morphological descriptions: Basidiomata 5–40 (−100) cm long, 1.5–12 cm thick, perennial, flat to slightly hoofed, solitary to imbricate, surface wavy to concentrically uneven, brown to dark brown, thin dull crust; Margin thin, white, Hymenophore whitish to ochraceous and brown, darker when touched. Pores round 4–6 per mm, dissepiments thick, entire. Tube layer dark brown, stratified, up to 13 cm thick. Context purplish brown, brighter than *G. adspersum*, corky, mottled with white patches; Hyphal system trimitic: generative hyphae with clamps 2–4 µm, colorless, branched, thin-walled; vegetative arboriform hyphae 4–5 (–6) µm wide, abundantly branched at the top, yellow-brown, aseptate, tapering ends; binding hyphae few; Cystidia absent; Basidia 20–35 × 8–10 µm, colorless, broadly club-shaped, 4 sterigmatic with a basal clamp; Basidiospores 7–8 (–9) × (4.5–) 5–6 (–6.8) µm ovoid-ellipsoid, truncated, ornamented, pale brown to reddish [28,99].

Distribution and habitat: Occurs in the temperate zone of Eurasia mainly on broadleaved trees, and more rarely on conifer trees [33,230].

Edibility, aroma, and flavor: Inedible, no significant odor and bitter taste.

#### 3.5.9. *Grifola frondosa* (Dicks.) Gray, a Natural Arrangement of British Plants 1: 643 (Table 3)

Ethnomedicinal uses: *G. frondosa* also known as hen-of-the-woods and maitake was a highly valued commodity in feudal Japan, with local lords exchanging it for an equivalent weight in silver. Thus, the Japanese name “dancing mushroom” comes from commoners in Japan who would dance for joy when they discovered *G. frondosa*, knowing they would be richly rewarded for their discovery. The mushroom was so highly valued in Japan, and the expert mushroom foragers would keep their harvest areas so secret that they would only reveal their locations after their death in their wills [163]. Based on literature data, in mainland Japan, China, and some other Asian countries, *G. frondosa* was popularly consumed for centuries as traditional medicine as curative herbal medication or health foods based on its enticing taste [18,164,231,232].

Medicinal uses: Many studies confirmed a broad spectrum of biological activities of *G. frondosa*, including anti-aging [164], antiallergic [160], antidepressant [157], antidiabetic [156,233], anti-inflammatory [160], antimicrobial [159,165], antioxidant [164,166], antitumor [159,160,163,225,234], antiviral [235], hepatoprotective [166], hypoglycemic [161], hypolipidemic [236], immunomodulatory and immunostimulatory [158,237], nephroprotective [161], and neuroprotective [16,158,160,163].

Morphological descriptions: Basidiomata annual, large, compound, multipileate, stipitate, and entire structure up to 15–60 cm wide. Pilei kidney-shaped, spatulate, petaloid to fan-shaped, flat to often curved, in imbricated and confluent clusters, each pileus up to 4–10 cm wide, smooth or radially glabrous, azonate or sometimes lightly zoned or wrinkled, pale lavender-grey, grayish-brown or pale brown at first to dark brown with age. Margin concolorous, thin, wavy, and sometimes lobed. Hymenium white, ivory-white to cream. Pores angular, slightly stretched and lacerated, 2–4 per mm, with thin and lacerated dissepiments. Tube layer decurrent on the stipe, distinct from context, brittle when dry, whitish or darker with age or when dry, up to 5 mm thick. Context ivory-white, firm, slightly fibrous, flexible, and 2 to 5 mm thick in individual pilei. Main stem branches and the base of stipitates can be several centimeters thick. A hypogeal structure called a sclerotium gives rise to a whitish, repeatedly branched stipe that can be up to 10 cm in diameter at the base. Hyphal system dimitic; generative hyphae curly, thin-walled, rarely branching, colorless, 2.5–5 µm in diameter; skeletal hyphae from context thick-walled, sparsely branched, 2.5–6 µm in diameter. Cystidia and other sterile hymenial elements are absent. Basidia clavate, with 4 sterigmata, clamped at the base, 20–35 × 5–8 µm. Basidiospores are ovoid to ellipsoid, smooth, thin-walled, often guttulate, colorless, 5.5–7 × 4–4.5 μm [215].

Distribution and habitat: This fungus is distributed in the temperate zone of the Northern Hemisphere, temperate forests in eastern North America, Europe, and Asia [163,238]. The most common substrate is *Quercus* spp. but may also occur on other hardwoods and occasionally on conifers [33,102,215].

Edibility, aroma, and flavor: Edible of choice when young. Tough and requiring long cooking when ripe. Pleasant, fragrant, nutty flavor, persistent and succulent, but slightly acidic, more pronounced when ripe.

#### 3.5.10. *Inonotus hispidus* (Bull.) P. Karst., Meddelanden af Societas pro Fauna et Flora Fennica 5: 39 (1879) (Table 3; Figure 5A)

Ethnomedicinal uses: *Inonotus hispidus* commonly known as the shaggy bracket is used in traditional medicine to treat dyspepsia, cancer, and diabetes. It is also used to treat digestive disorders (parasites, diarrhea, general internal cleaning) as well as diseases of the heart, liver, stomach, and abdominal pain. Furthermore, when used for cleansing the genitals during menstruation and for newborns, the infusion of *I. hispidus* exhibits antiseptic properties [171]. *I. hispidus* is reported to be used as ancient medicinal materials and health care products in Chinese traditional medicine. It is used as a diuretic, an astringent, and to treat canker sores and inflammation in folk culture [167].

Medicinal uses: Numerous studies have confirmed the anti-inflammatory, antifungal, antimicrobial, antioxidant, and antiviral properties, as well as antitumor, immunomodulatory, immunostimulatory, antiproliferative, and cytotoxic effects of I. hispidus extracts [168]. Studies have shown promising immunomodulatory activity of extracts from the *I. hispidus* basidiomes, with presence of the bioactive substances such as hispolon and hispidin [170]: natural killer T cell activity and function increased with a dose of mushroom fruiting body extract. Later, it is concluded that *I. hispidus* may be a new source of neurotrophic and protective agents against neurodegenerative diseases [172]. Neurodegenerative diseases, which mainly affect the elderly, include Alzheimer’s, Parkinson’s, Huntington’s, and Pick’s disease, and are characterized by the slow death of specific groups of nerve cells and increasing atrophy of the corresponding parts of the brain or spinal cord [169]. In a recent study, Zhang et al. [173] discovered that I. hispidus demonstrates hypolipidemic effects in laboratory mice by effectively suppressing inflammation caused by oxidative stress.

Morphological descriptions: Basidiomes annual, sessile, applanate, dimidiate, solitary rarely imbricate, effused up to 30 cm long and 8 cm thick; hispid, hirsute to rarely strigose, azonate, rough, watery, spongy, soft when fresh, reddish-orange when young, dark reddish brown to dark brown to blackish in the mature stage. Margin obtuse, concolorous with pilear surface; Pore surface olivaceus yellow becoming brown to blackish-brown. Pores angular, 1–3 per mm, but often wider with thin and lacerate dissepiments. Large holes irregularly placed among pores; 2–4 mm wide. Context azonate, spongy, hygrophanous, fleshy when fresh, brittle when dry, reddish brown to dark brown, up to 5 cm thick; it turns black when exposed to potassium hydroxide (KOH); Tube yellowish brown to dark brown, up to 10–20 mm thick. Hypha monomitic, generative hyphae yellowish brown, simple-septate, thin-walled, rarely branched, interwoven but with a parallel arrangement in the subhymenium, 2.5–3.9 μm wide; contextual hyphae thin-walled, pale yellowish-brown, rarely branched and septate, up to 4.3–9.5 μm wide; tramal hyphae brown, branched and thick-walled, 4–6 μm wide; some hyphae yellow brown, branched almost with solid walls, with a strong tortuosus, twisted arrangement, up to 10–12 μm wide; Hyphae of dissepiments hyaline, thin-walled, apically rounded, long-celled, septate; hispid hyphae of hispid epicutis brown, thin-walled, tightly packed forming hyphal strings, interwoven but with a parallel arrangement, up to 6 μm wide; hymenial setae brownish dark brown, rare to abundant, thick-walled, ventricose, with an acute and hooked apex, 20.9–33.6 × 8.7–11 μm; Basidia hyaline, broadly clavate, with a simple basal septum, 28–33 × 10–12 μm; Basidiospores smooth, subglobose, brown, guttulate, thick-walled, 8.0–9.8 (–10.2) × (7.2–)7.5–8.8(–9.2) μm [99].

**Figure 5:**
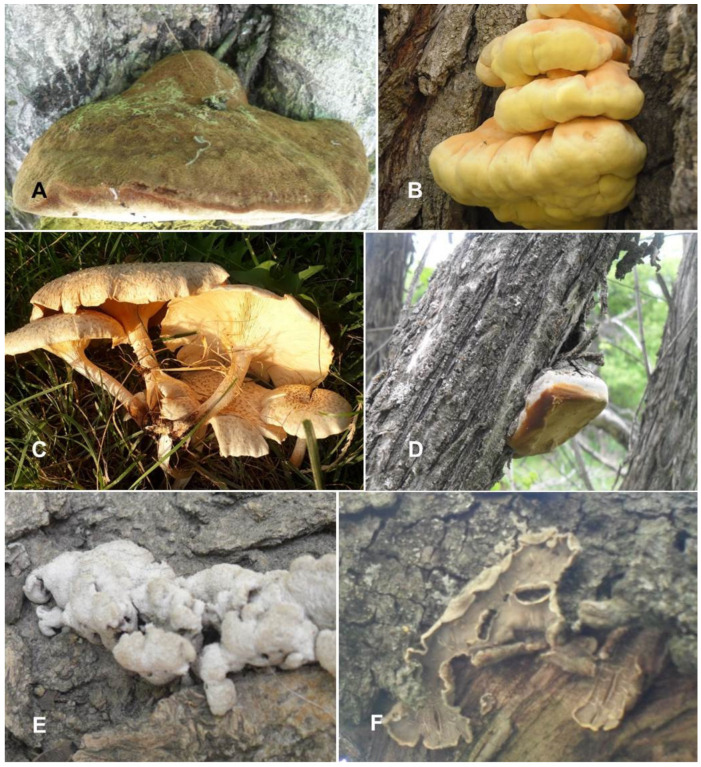
*Inonotus hispidus* (**A**), *Laetiporus sulphureus* (**B**), *Lentinus tigrinus* (**C**), *Sanghuangporus lonicerinus* (**D**), *Schizophyllum commune* (**E**), *Stereum hirsutum* (**F**), Photo credit by Yusufjon Gafforov.

Distribution and habitat: Grows on living *Quercus* spp. but also on a very large number of angiosperms woody plants. Common and widespread species that can be found in gardens, parks, orchards, and forests. Europe and Asia have a temperate distribution [33,99,220].

Edibility, aroma, and flavor: Inedible, aroma and flavor are irrelevant.

#### 3.5.11. *Laetiporus sulphureus* (Bull.) Murrill, Mycologia 12 (1): 11 (1920) (Table 3; Figure 5B)

Ethnomedicinal uses: Its common names are crab-of-the-woods, sulfur polypore, sulfur shelf, and chicken-of-the-woods. According to Icones of Medical Fungi from China [114], *L. sulphureus* fruit bodies are thought to be capable of regulating the human body, improving health, and defending the body against illnesses; it has also been used to repel mosquitoes and midges by burning fruit bodies [114]. *L. sulphureus* has long been used in Asian herbal medicine [239]. In folk medicine, it is used in powders, water, and alcohol tinctures. It is widely used in the treatment of diseases such as pyretic diseases, coughs, gastric cancer, and rheumatism [17].

Medicinal uses: This fungus is known as a source of antimicrobial, antitumor, anti-inflammatory, anticoagulant, antioxidant, cytostatic, hypoglycemic, and immunostimulant agents as well as a producer of HIV-1 reverse transcriptase inhibitors [174,239]. Due to their long history of medicinal uses, the biologically active compounds and extracts from *L. sulphureus* exhibit a broad spectrum of pharmacological activities as anti-diabetic, anti-malarial, anti-thrombin, anti-ulcer, antiviral, hepatoprotective, and immunomodulating [17,175,240].

Morphological descriptions: Basidiomata annual, sessile to substipitate, pilei single or growing in large clusters up to 50 cm in diameter. Pilei semicircular to fan-shaped, 5–25 cm in long and up to 20 cm wide; up to 3 cm thick; flat to convex; Surface undulate, smooth or finely wrinkled; bright yellow to orange when fresh, fading to pale yellow or brownish when old and dry. Margin thick, undulate, rounded, concolorous with upper surface. Hymenophore sulfur yellow to yellow ochraceous, pores round to angular, 3–4 per mm, with thin and entire dissepiments, quickly becoming lacerate. Tube layer up to 5 mm deep, yellow. Context yellowish or white, azonate, watery, fleshy, and sappy when fresh and chalky, crumbly and brittle when dry, up to 2 cm thick. Hyphal system dimitic: generative hyphae rarely apparent, simple-septate, thin-walled, branched, colorless, 6–12 µm in diameter; contextual binding hyphae branched and interlocked, nonseptate, firm to thick-walled, colorless, 3–20 µm wide, dominant in the context tissue; tramal hyphae more parallel than those in context; regularly septate-simple, thin-walled, colorless 3–7 µm in diameter. Cystidia and other sterile hymenial elements are absent. Basidia clavate, thin-walled, with 4 sterigmata, simple-septate at the base, 18–25 × 6–9 µm. Basidiospores are broadly ellipsoid to ovoid, smooth, thin-walled, colorless, 5.4–7.5 × (3.5)4–6 µm [99].

Distribution and habitat: *L. sulphureus* has been treated as a cosmopolitan species present on all continents except Antarctica, from boreal to subtropical and tropical zones [241]. The most common hosts of *L. sulphureus* in Europe are hardwoods such as *Quercus*, *Fagus*, *Populus*, *Prunus*, *Pyrus*, *Robinia*, and *Salix*, and rarely also conifers such as *Cupressus* or *Taxus* [33,99,220]. *L. sulphureus* was recorded on *Acacia*, *Acer*, *Juglans*, *Prunus*, *Robinia*, and *Quercus* in Uzbekistan [33].

Edibility, aroma, and flavor: Edible when young, strong odor and pleasant taste.

#### 3.5.12. *Lentinus tigrinus* (Bull.) Fr., Systema Orbis Vegetabilis 1: 78 (1825) (Table 3; Figure 5C)

Ethnomedicinal uses: *L. tigrinus* (Tiger Sawgill) is widely used as food, medicine, brain tonic, and against anger. Powder taken in hot water relieves dry cough and asthma; the raw materials are also useful for wet cough. Mixing raw fruit bodies with lemon juice improves gastrointestinal function and enhances digestion [177]. Indigenous communities considered this mushroom as a potential source of antibacterial drugs and as remedies for arthritis, cough and colds, fever, headache, hypertension, skin diseases, stomachaches, and toothaches. They usually boil or grind the mushroom to obtain an extract; they drink the broth or apply the fungus directly to infected body parts [183].

Medicinal and environmental uses: This mushroom has antimicrobial and antioxidant [179], hypoglycemic and antidiabetic [176], anti-inflammatory [178], anticancer [181,182], and immunomodulatory properties [180]. In addition, Mohammadnejad et al. [181] reported the anticancer potentiality of a soluble protein fraction of *L. tigrinus* that showed greater antiproliferative and cytotoxic activities against PC3 cells; this suggests that the soluble protein fraction of this mushroom may be considered a potent anticancer compound. Recently, Pourianfar et al. [182] found that wild Iranian *L. tigrinus* strain may be regarded as a source of macro- and micronutrients with specific anticancer potential against MCF-7 cells; however, it poses little risk to humans when consumed in small amounts.

Morphological descriptions: Basidiomata annual, pileate with a central stipe, single or in groups. Pileus 4–10 cm wide, fleshy-skinny, convex, later depressed in the center or funnel-shaped, Upper surface dry, white, slightly yellowish, creamy, covered with dark brown. Margin acute, undulating, and split in age. Hymenophores are cream-colored to yellowish, lamellae decurrent, crowded, with slightly jagged edges; short lamellulae frequent. The flesh is tough, dense, whitish, thin, whitish, and sometimes turns yellow with age. Stipe: central or eccentric, cylindrical, dense, rigid, even or curved, narrowed towards the base, cream or whitish, with small concentric, brownish, sparse scales, sometimes with annular zone when young, 2–5 cm long and 4–8 mm thick, root-like elongated base often immersed in wood. Hyphal system dimitic: generative hyphae 2.5–8 µm wide, with buckles, moderately branching in the stipe and tissue of the pileus, in the cuticle radially located colorless or with thickened brownish walls, strongly swollen (up to 8 µm in diameter). Skeletal hyphae 2–5 µm in diameter, colorless, predominate in the stem. Cheilocystidia similar to basidioles 30–50 × 3–7.5 µm, cylindrical to almost club-shaped, thick-walled, hyaline. Fusoid cystidioles are 20–25 × 2–3 µm, slenderly clavate, 4 sterigmata, 20–30 × 4–5.5 µm, with a basal clamp. Basidiospores 6–8 × 2.7–3.5 µm; cylindrical to ellipsoid, smooth [242].

Distribution and habitat: Distributed in the Northern Hemisphere, Eurasia, and North America. In Central Asia, it is common in forests, gardens, and along roadsides, especially where walnuts, poplars, and willows grow [33,102]. This fungus occurs on hardwood, usually in floodplain regularly moistened forests. It grows mainly on *Salix* and *Populus*, less common on other hardwoods.

Edibility, aroma, and flavor: Edible, fruity, pleasant odor, and pleasant mushroom but later astringent flavor.

#### 3.5.13. *Phellinus igniarius* (L.) Quél., Enchiridion Fungorum in Europa Media et Praesertim in Gallia Vigentium: 172 (1886) (Table 3)

Ethnomedicinal uses: The fruiting bodies of *P. igniarius* (Willow Bracket) have been historically used as a folk medicine for the treatment of endometrorrhagia in gynecology [188]. This species has the ability to scavenge free radicals and prevent other diseases in traditional medicine; it would reduce the risk of heart attack, stroke, and cancer. Drinking its tinctures and broths is used as a treatment for various injuries, and inflammations [109]. *P. igniarius* was burned, mixed with tobacco, and chewed. The alkaline properties of ash are known to increase the penetration of nicotine into the bloodstream. The Arctic tribes boiled the pulp of the fruitbodies and drank it as a broth to relieve stomach ailments or pain. A tincture or tobacco prepared with this fungus has also been used to protect the liver in patients with liver damage due to alcohol abuse. *P. igniarius* tea is a very popular antioxidant, which is believed to inhibit cancer [184].

Medicinal uses: This fungus is rich in biologically active substances with therapeutic potential. Mycochemical studies have proved the presence of polysaccharides, phenolic compounds, and terpenoids. These compounds showed biological activities such as antiangiogenic, anticancer, antioxidant, and antiviral. Research studies conducted using modern analytical methods have advanced the knowledge of the potential therapeutic uses of compounds isolated not only from the fruiting bodies but also from biomass obtained with in vitro biotechnological methods [148,187,188,189].

Morphological descriptions: Basidiomata perennial, small to large in size, up to 25 cm in diameter and 15 cm thick, hard, woody, first nodulose, then ungulate or triangular in section. Surface crusty, brown, grey, to grey-black concentrically sulcate, deeply cracked. Margins blunt to rounded, yellowish, brown to grey. Hymenophore poroid brown, cinnamon brown, reddish-brown. Pores roundish, 4–6 per mm, dissepiments entire, variable in thickness. Context zonate, woody, very hard, reddish brown, or cinnamon brown, up to 3 cm thick. Tube layers are stratified and concolorous with context including white mycelium, each layer 4 mm thick. Cystidia absent. Hymenial setae, ventricose to subulate 12–20 × 4.5–9 µm, thick-walled, reddish brown. Hyphal system dimitic: generative hyphae thin-walled, 2–3 µm wide with simple septa. Skeletal hyphae brown, aseptate, thick-walled, 2–5 µm wide. Basidia are broadly clavate, 4-spored, 8–12 × 6–7 µm, with simple septa at the base. Basidiospores subglobose to broadly ovoid, 5–6.5 × 4.5–6 µm, thick-walled [99].

Distribution and habitat: The fungus found on hardwood living and later dead trees. *P. igniarius* in the narrow sense appears to occur in Uzbekistan and other Central Asian countries; it is recorded on *Acer*, *Juglans*, *Lonicera*, and *Salix* [33].

Edibility, aroma and flavor: Inedible

#### 3.5.14. *Sanghuangporus lonicerinus* (Bondartsev) Sheng H. Wu, L.W. Zhou and Y.C. Dai, Fungal Diversity 77: 340 (2015) (Table 3; Figure 5D)

Ethnomedicinal uses: Recently, Zhou et al. [191] defined “Sanghuang” as one of the most important groups of medicinal mushrooms with fourteen described species in the genus *Sanghuangporus* including *S. lonicerinus*, which have been used in TCM for the past two centuries. Wu et al. [190] reported *S. lonicerinus* is commonly used in Hubei and other places, as well as by the Tujia ethnic minority. All species in Sanghuangporus can be considered to be medicinal macrofungi [190,191].

Medicinal uses: Based on modern pharmacological studies, *S. lonicerinus* was reported to have multifaceted biological activities, including antiproliferative [186], and hepatoprotective effects [185]. Shen et al. [243] recently reported widespread misidentifications of traditional medicinal mushrooms in the *Sanghuangporus* genus via ITS barcoding and designation of reference sequences.

Morphological descriptions: Basidiomata perennial, pileate, dimidiate, single, rarely fused, up to 15 cm wide, 10 cm long, and 8 cm thick. Upper surface dark brown to black, glabrous, heavily cracking rimose, margin concolorous, thick, blunt. Hymenophore poroid, pore surface sulphury yellow to golden brown; pores 4–5 per mm, circular; dissepiments entire. Context light brown, tough, up to 2.5 cm thick. Tube layers are concolorous with context, up to 2.5 cm long. Hyphal system monomitic in context, dimitic in trama: generative hyphae simple-septate, colorless to golden brown, thin to thick-walled, moderately branched, 3–4 μm wide; skeletal hyphae golden brown, thick-walled, 2–3.5 μm in diameter. Cystidia absent, hymenial setae ventricose, thick-walled, 10–22 × 5–7(–8) μm. Basidia are 4 sterigmata and 7–11 × 4–5 μm. Basidiospores are light yellow, broadly ellipsoid, 4.3–5 × 3.3–3.8 μm, slightly thick-walled, smooth, cyanophilous, and negative in Melzer’s reagent [69,244].

Distribution and habitat: *S. lonicerinus* is distributed throughout the republics of Central Asia, Iran, and Russia and is reported on *Lonicera* spp. [33,102].

Edibility, aroma, and flavor: Not distinctive, inedible.

#### 3.5.15. *Schizophyllum commune* Fr., Observationes mycologicæ 1: 103 (1815) (Table 3; Figure 5E)

Ethnomedicinal uses: *S. commune* (Common split gill) has been renowned in Far Eastern countries as medicine for hundreds of years [192]. In relation to this, it has been used in TCM in the form of infusions to treat leucorrhea and to regulate blood pressure [245]. Milenge Kamalebo et al. [246] claimed that people used *S. commune* in the treatment of breast inflammation and wounds.

Medicinal uses: *S. commune* is a potent and well-studied edible medicinal mushroom with analgesic [195], antidiabetic [197,247], anti-inflammatory [194], antimicrobial [198,199,248], antioxidant [193,196,197,199], antitumor, chemoprotective, immunostimulating [240], and neuroprotective [199] effects. In this regard, Umeo et al. [249] mentioned that S. commune is an important producer of hydrolytic enzymes with biotechnological potential, antioxidant, and immunomodulatory activities and is also used in the form of antitumor polysaccharide schizophyllan (SPG) as an adjuvant treatment for tumors. Garcia et al. [250] reviewed that the most promising effects of SPG seem to be antitumor and immunomodulation actions, based on its clinical use for the treatment of several cancers in Japan. Vu et al. [201] recently demonstrated that *S. commune* β-glucan improved intestinal health by enhancing water absorption, proliferating epithelial cells, and increasing lubricating mucin production, which is essential for the treatment of constipation. Moreover, the administration of β-glucan prevents the risk of atherosclerosis, diabetes, intestinal inflammation diseases, and obesity based on the reduction in serum glucose levels and biomarkers associated with liver injury and up-regulating of HDL [201]. In addition, Zeynali et al. [202] reported the antibacterial activity and healing properties of a dressing made from an *S. commune*-derived chitin glucan complex using an animal model of second-degree burn. These nanofibers exhibited biocompatibility, as they do not cause toxicity to fibroblast cells and, in fact, enhance their proliferation and adhesion capabilities and potentially could be utilized as wound dressings.

Morphological descriptions: Basidiome pileate, kidney-shaped, conchate, fan-shaped, sessile or attached with a short stipe, 2–3(4) cm in size. Upper surface felty, pubescent to strigose, with concentric zones and longitudinal whitish, pinkish, or greyish wrinkles of varying prominence. Hymenophore is distinguished by lamellae that grow radially from the base and split on the edges when mature. Lamellae become cream, pinkish-grey, yellowish-gray, or brown as they age. The edges are wavy, even, or lobed, becoming hardened in age; split when dry, and closed again when wet. The flesh is thin, dense, soft to leathery when fresh, firm, and tough when dry. Hyphal system pseudodymitic, 1.5–7 µm in diameter, with simple septa or occasional clamps. sometimes stuck together. Basidia 40–55 × 7–10 µm, slenderly clavate, 4 sterigmate with a basal clamp; Basidiospores are 5.5–79 × 1.5–2.5(3.0) µm, cylindric and slightly curved, smooth, thin-walled, colorless, and with drops [242].

Distribution and habitat: This species is a cosmopolitan species that colonize living trees and dead wood of broadleaved trees, rarely conifers. In Uzbekistan, this fungus is found on *Celtis caucasica*, *Juglans regia*, *Malus domestica*, *Morus alba*, *Populus* sp., *Prunus armeniaca*, and *Salix pentandra* Ehrh. [33].

Edibility, aroma, and flavor: Not distinctive. But in youth, the common slit-leaf has tender flesh and excellent taste. In European and American countries, it is considered inedible, largely due to cultural tastes, small stature, and its presumed leathery texture. Literature reports on *S. commune* have mainly implicated the fungus with multiple pulmonary manifestations ranging from sinusitis, allergic bronchopulmonary mycoses, asthma, chronic eosinophilic pneumonia, and so on [251,252].

#### 3.5.16. *Stereum hirsutum* (Willd.) Pers., Observationes Mycologicae 2: 90 (1800) (Table 3; Figure 5F)

Ethnomedicinal uses: *Stereum hirsutum* called hairy curtain crust fungus has been used as a food and folk medicine in Chinese society [204,205]. It has been used in traditional medicine to treat diabetes and dyspepsia. It is also applied in India as an antiseptic [203]. Mišković et al. [207] mentioned that the fruiting bodies of *S. hirsutum* were used as traditional folk medicine in China and Korea for cancer treatment.

Medicinal uses: Duan et al. [204] summarized that the fermented mycelia of *S. hirsutum* produced biologically active secondary metabolites, such as antibacterial epidioxysterols, benzoate derivatives, bioactive vibralactone, multiple active sesquiterpenoids, and phytotoxic active acetylenic compounds among others. In summary, most of them showed a broad diversity of biological activities including antibacterial, antifungal, anti-nematocidal, antiviral, autophagy-inducing activity, pancreatic lipase inhibition, and antiparasitic in malaria [206], as well as antidiabetic, antimicrobial, antioxidant, cytotoxic, and neuroprotective activities, among others [200,207,208].

Morphological descriptions: Basidiome perennial, tough to hard, resupinate, effuse-reflexed to pileate, semicircular to flabelliform, a few centimeters long, up to 2 mm thick, and with pilei projecting 5–30 mm, Basidiomata are also known as basidiophytes. Due to the presence of algae, the upper surface is tomentose to hirsute, zonate, whitish to yellowish orange, and later brown and greenish. Lighter and undulating margin. Smooth to slightly tuberculate, occasionally cracking when dry, yellowish orange, and grayish-brown when old is the hymenophore. Context yellowish ochraceous up to 1 mm thick, separated from the tomentum by a thick dark layer. Consistency is elastic and tough when fresh, and brittle and hard when dry. Hyphal system pseudodimitic: generative hyphae thin to thick-walled, 2–3 µm wide, septate, colorless; pseudo skeletal hyphae thick-walled, without septa, 3–6 µm wide; All hyphae strongly glued together. Cystidial elements of two kinds: Pseudocystidia emerging from skeletal hyphae, obtuse to acute mostly thick-walled very long (more than 100 µm long, 7–10 µm wide), colorless to yellowish; acutocystidia pointed 20–30 × 2–4 µm. Basidia are slender, with 4 sterigmata, 25–50 × 3–5 µm, and simple-septate at the base. Basidiospores narrowly ellipsoid to cylindrical, thin-walled, smooth, colorless, 5–6.5 (–8) × 2–3.5 µm, amyloid [99].

Distribution and habitat: Widespread in the northern and southern hemispheres [253], usually in the temperate zone. This species grows on dead trunks and branches, and also on dead branches and exposed dead wood of living trees. In Uzbekistan, *Stereum hirsutum* was recorded on eight tree genera: *Acer*, *Celtis*, *Crataegus*, *Fraxinus*, *Juglans*, *Populus*, *Quercus*, and *Salix* [33].

Edibility, aroma, and flavor: Inedible, no special smell or taste.

#### 3.5.17. *Trametes versicolor* (L.) Lloyd, Mycol. Writ. 6 (65): 1045 (1920) (Table 3)

Ethnomedicinal uses: *Trametes versicolor*, the Turkey tail fungus (formerly known as the Many-Zoned Polypore), is one of the best well-known traditional medicinal mushrooms used in China for over 2000 years [211]. In TCM, this fungus has been used as a tea or soup to treat cancers of the liver, jaundice, fever, spleen as well as heart, stomach, and sometimes kidney disorders [254]. It was also used as a tonic for the regulation of asthma, coughs, immune functions, and in the case of increased appetite [254]. *T. versicolor* was frequently used in TCM for its immunomodulatory and antitumor activities [255]. It was considered useful in dispelling fever, improving immune functions, eliminating toxins, and strengthening the physical health of the patient [209]. On the other hand, in the clinical practices of TCM, *T. versicolor* was often indicated for various types of cancer, chronic hepatitis, and infections of the upper respiratory, urinary, and digestive tracts [209,256].

Medicinal uses: *T. versicolor*, an important non-edible medicinal species, has gained widespread popularity due to its broad spectrum of beneficial properties including anti-acetylcholinesterase, antidiabetic, anticoagulant, anti-inflammatory, antimicrobial, anti-obesity, antioxidant, antitumor, cardioprotective, hepatoprotective, hypocholesterolemic and hypolipidemic activities as well as immunoregulatory and immunostimulatory effects [210,212,213]. In addition, it is the most commonly used fungus for various cancers affecting the breast, cervix, lungs, esophagus, and uterus, as well as when leukemia, lymphoma (lymphatic tissue that causes swollen nodes), melanoma (skin cancer), and brain cancer. Recently, He et al. [188] isolated musarin, a novel protein from *T. versicolor* that has shown potency to be developed as a promising new therapeutic drug candidate to support colorectal cancer treatment.

Morphological descriptions: Basidiome annual, sessile or effused-reflexed, fan-shaped to spathulate, up to 10 cm long and 8 mm thick. Pilei form numerous groups, coriaceous, laterally fused or imbricate in clusters, tough and elastic. The upper surface is velvety, tomentose to hirsute, and highly variable in colors including cream, ochraceous, reddish brown, and brown, blue, gray to almost black or greenish due to microscopic algae. The margin is thin, lobed, and velvety. Hymenophore white to cream or ochraceous pores round-angular 4–5 per mm with entire and thick dissepiments. Tube layer white to cream, up to 3 mm thick. Context: thin black line separating the tomentum from the tough, cream-colored, 1–4 mm thick object. Hyphal system trimitic: contextual binding hyphae thick-walled and aseptate, unbranched, 2.5–8 m wide; contextual skeletal hyphae thick-walled and aseptate, unbranched, 1.5–3 m wide; generative hyphae colorless, thin-walled, branched with clamps. Fusoid cystidioles are present but there are no cystidia. They are thin-walled, measuring 12–19–4–5 m. Basidia clavate, 15–20 × 4–6 mm, with a basal clamp and 4 sterigmata. Basidiospores are colorless, cylindrical, slightly curved, and 5–7 × 1.8–2.5 µm [99].

Distribution and habitat: The species has a global distribution and is widespread in temperate Eurasia [214]. This species is commonly found on weakened or wounded living trees, dead trees, dead large branches, and stumps. In Central Asia, it occurs on *Betula*, *Crataegus*, *Celtis*, *Juglans*, *Lonicera*, *Malus*, *Prunus*, and *Quercus* trees [33].

Edibility, aroma, and flavor: Inedible, without odor and taste.

## 4. Conclusions

Firstly, the present investigation focuses on the diversity of macrofungi collected from several provinces of Uzbekistan in 2021–2022 using morphological characters and molecular data. Seventeen macrofungal species of basidiomycetous from 11 families and 16 genera have been identified, possessing edible and/or medicinal properties. Ethnomedicinal and medicinal applications, morphological characteristics, molecular phylogeny, host preferences, geographical distribution, and habitat of the collected macrofungi have been provided.

Secondly, our study is the first to focus on the ethnomycological diversity and ethnomedicinal uses of medicinal fungi from Uzbekistan, knowing that basidiomycete macrofungi are ecologically and economically pathogenic, nutritional, and medicinal resources. In the provinces of Uzbekistan, 17 species of basidiomycetous macrofungi of great ethnomycological importance from ten families and sixteen genera have been identified, possessing edible and/or medicinal properties.

A comprehensive overview of ethnomycological knowledge, traditional and modern medicinal uses, and pharmacological data of macrofungal species has been developed. This information serves as valuable scientific information on the use of medicinal mushrooms in folk and modern medicine across the country. These reports constitute effective scientific data on the various uses of medicinal mushrooms in folk and modern medicine in Uzbekistan, with a broad spectrum of applications.

Finally, the information described in this work can be used to better utilize the medicinal value of medicinal mushrooms growing in Uzbekistan. In addition to highlighting their use in medicine, it should also be recognized that the creation of a national database of macrofungi in Uzbekistan is becoming a topical issue today. Moreover, it is to be recommended that they be studied phytochemically with the latest pharmaceutical procedures that can eventually lead to drug discovery.

## Figures and Tables

**Table 1:** Description of the study sites.

Location	Altitude (m)	Locality	Forest Type	Habitat
Andijan Prov.	1450	40.7686° N, 72.2364° E	Flood-plain and urban forest	Several trees (*Salix* sp., *Populus* spp., *Malus* spp., *Prunus* spp., *Juglans* sp., *Vitis* spp., and *Morus* spp.)
Fergana Prov.	400–1400	40.4568° N, 71.2874° E	Desert, flood-plain, urban and mountain forests	Trees and shrubs forests (*Haloxylon* spp., *Tamarix* spp., *Populus* spp., *Salix* spp., *Rosa* spp., *Malus* spp., *Prunus* spp., *Lonicera* spp., and *Speria* spp.)
Jizzakh Prov.	365–2000	40.4706° N, 67.5709° E	Zaamin State Reserve and Zaamin National Park	Juniper forest (*Juniperus* spp., *Lonicera* spp., and *Prunus* spp.)
Namangan Prov.	432–1600	41.0510° N, 71.0973° E	Urban and Mountain Forest	Trees and shrubs forests (*Populus* spp., *Salix* spp., *Rosa* spp., *Malus* spp., and *Prunus* spp.)
Samarkand Prov.	577–800	39.9208° N, 66.4271° E	Mountain Forest	Trees and shrubs mixed forests (*Juniperus* spp., *Rosa* spp., *Lonicera* spp., *Crataegus* spp., and *Sorbus* spp.)
Tashkent Botanical Garden	470–500	41.3448° N, 69.3107° E	Botanical garden	Several introduced trees and shrubs
Tashkent Prov.	1100–1800	41.2213° N, 69.8597° E	Ugam-Chatkal State National Natural Park	Trees and shrubs mixed forests (*Juniperus* spp., *Crataegus* spp., *Lonicera* spp., *Salix* spp., *Juglans* spp., *Malus* spp., *Prunus* spp., and *Fraxinus* spp.)

## Data Availability

The DNA sequence data obtained from this study were deposited in GenBank under the accession numbers ITS (OR250340-OR250363).

## Abbreviations

SPG	Schizophyllan
TCM	Traditional Chinese Medicine

## References

[1] Sitotaw R., Lulekal E., Abate D. (2020). Ethnomycological study of edible and medicinal mushrooms in Menge District, Asossa Zone, Benshangul Gumuz Region, Ethiopia. J. Ethnobiol. Ethnomed..

[2] Bautista-González J.A., Montoya A., Bye R., Esqueda M., Herrera-Campos M.D. (2022). Traditional knowledge of medicinal mushrooms and lichens of Yuman peoples in Northern Mexico. J. Ethnobiol. Ethnomed..

[3] Sharma R., Sharma Y.P., Hashmi S.A., Kumar S., Manhas R.K. (2022). Ethnomycological study of wild edible and medicinal mushrooms in district Jammu, J&K (UT). India. J. Ethnobiol. Ethnomed..

[4] Ullah T.S., Firdous S.S., Shier W.T., Hussain J., Shaheen H., Usman M., Akram M., Khalid A.N. (2022). Diversity and ethnomycological importance of mushrooms from Western Himalayas, Kashmir. J. Ethnobiol. Ethnomed..

[5] Wang R., Herrera M., Xu W., Zhang P., Moreno J.P., Colinas C., Yu F. (2022). Ethnomycological study on wild mushrooms in Pu’er Prefecture, Southwest Yunnan, China. J. Ethnobiol. Ethnomed..

[6] Wasser S.P. (2010). Medicinal mushroom science: History, current status, future trends, and unsolved problems. Int. J. Med. Mushrooms.

[7] Du J., Li X., Yang H., Teng S., Liu J., Feng T. (2023). Phellinilludins A–D, new illudane sesquiterpenes from the fungus *Phellinus tremulae*. Phytochem. Lett..

[8] Galappaththi M.C.A., Patabendige N.M., Premarathne B.M., Hapuarachchi K.K., Tibpromma S., Dai D.Q., Suwannarach N., Rapior S., Karunarathna S.C. (2022). A review of *Ganoderma* triterpenoids and their bioactivities. Biomolecules.

[9] Rašeta M., Karaman M., Jakšić M., Šibul F., Kebert M., Novaković A., Popović M. (2016). Mineral composition, antioxidant and cytotoxic biopotentials of wild-growing *Ganoderma* species (Serbia): *G. lucidum* (Curtis) P. Karst vs. G. applanatum (Pers.) Pat. Int. J. Food Sci. Technol..

[10] Steimbach L.M., Borgmann A.V., Gomar G.G., Hoffmann L.V., Rutckeviski R., de Andrade D.P., Smiderle F.R. (2021). Fungal beta-glucans as adjuvants for treating cancer patients—A systematic review of clinical trials. Clin. Nutr..

[11] Karaman M., Čapelja E., Rašeta M., Rakić M., Arya A., Rusevska K. (2022). Diversity, Chemistry, and Environmental Contamination of Wild Growing Medicinal Mushroom Species as Sources of Biologically Active Substances (Antioxidants, Anti-Diabetics, and AChE Inhibitors). Biology, Cultivation and Applications of Mushrooms.

[12] Khatua S., Acharya K. (2022). Antioxidation and immune-stimulatory actions of cold alkali extracted polysaccharide fraction from *Macrocybe lobayensis*, a wild edible mushroom. 3 Biotech..

[13] Kolniak-Ostek J., Oszmianski J., Szyjka A., Moreira H., Barg E. (2022). Anticancer and antioxidant activities in *Ganoderma lucidum* wild mushrooms in Poland, as well as their phenolic and triterpenoid compounds. Int. J. Mol. Sci..

[14] Rosdan Bushra S.M., Nurul A.A. (2022). Bioactive mushroom polysaccharides: The structure, characterization and biological functions. J. Liq. Chromatogr. Relat. Technol..

[15] Vetter J. (2023). The mushroom glucans: Molecules of high biological and medicinal importance. Foods.

[16] Zhao J., He R., Zhong H., Liu S., Liu X., Hussain M., Sun P. (2023). A cold-water extracted polysaccharide-protein complex from *Grifola frondosa* exhibited anti-tumor activity via TLR4-NF-κB signaling activation and gut microbiota modification in H22 tumor-bearing mice. Int. J. Biol. Macromol..

[17] Khatua S., Ghosh S., Acharya K. (2017). *Laetiporus sulphureus* (Fr.) Murr. as food as medicine. Pharmacogn. J..

[18] Živković J., Ivanov M., Stojković D., Glamočlija J. (2021). Ethnomycological investigation in Serbia: Astonishing realm of mycomedicines and mycofood. J. Fungi.

[19] Campi M.G., Mancuello C., Maubet Y., Cristaldo E., Veloso B., Ferreira F., Thornton L., Robledo G. (2023). Biochemical, nutritional, and toxicological properties of the edible species *Phlebopus beniensis* with ethnomycological notes from Paraguay. Braz. J. Food Technol..

[20] See Toh C.J.Y., Bi X., Lee H.W., Yeo M.T.Y., Henry C.J. (2023). Is mushroom polysaccharide extract a better fat replacer than dried mushroom powder for food applications?. Front. Nutr..

[21] Wang L., Lian J., Zheng Q., Wang L., Wang Y., Yang D. (2023). Composition analysis and prebiotics properties of polysaccharides extracted from *Lepista sordida* submerged cultivation mycelium. Front. Microbiol..

[22] Gafforov Y. (2017). A preliminary checklist of ascomycetous microfungi from Southern Uzbekistan. Mycosphere.

[23] Gafforov Y., Rakhimov D. (2017). *Diplodia* and *Dothiorella* species (Botryosphaeriaceae, Ascomycota) from Uzbekistan. J. Bot. Res. Inst..

[24] Pem D., Gafforov Y., Jeewon R., Hongsanan S., Promputtha I., Doilom M., Hyde K.D. (2018). Multigene phylogeny coupled with morphological characterization reveal two new species of *Holmiella* and taxonomic insights within Patellariaceae. Cryptogam. Mycol..

[25] Pem D., Jeewon R., Gafforov Y., Hongsanan S., Phukhamsakda C., Promputtha I., Doilom M., Hyde K.D. (2019). *Melanocamarosporioides ugamica* gen. et sp. nov., a novel member of the family *Melanommataceae* from Uzbekistan. Mycol. Prog..

[26] Pem D., Jeewon J., Bulgakov T., Gafforov Y., Hongsanan S., Phookamsak R., Xu J., Promputtha I., Doilom M., Hyde K.D. (2019). Taxonomy and molecular phylogeny of *Thyrostroma ephedricola* sp. nov. (Dothidotthiaceae) and proposal for *Thyrostroma jaczewskii* comb. nov. Phytotaxa.

[27] Abdurazakov A.A., Bulgakov T.S., Kholmuradova T.N., Gafforov Y.S. (2021). Powdery mildew fungi (Erysiphaceae) of the Fergana Valley (within Uzbekistan): A first annotated checklist. Nov. Sist. Nizshikh Rastenii.

[28] Gafforov Y.S. (2014). Taxonomy and diversity of the genus *Ganoderma* Karst. (Basidiomycota) species in Uzbekistan. Uzb. Biol. J..

[29] Yatsiuk I., Saar I., Kalamees K., Sulaymonov S., Gafforov Y., O’Donnell K. (2016). Epitypification of *Morchella steppicola* (Morchellaceae, Pezizales), a morphologically, phylogenetically and biogeographically distinct member of the Esculenta Clade from central Eurasia. Phytotaxa.

[30] Gafforov Y., Tomšovský M., Langer E., Zhou L.W. (2014). *Phylloporia yuchengii* sp. nov. (Hymenochaetales, Basidiomycota) from Western Tien Shan Mountains of Uzbekistan based on phylogeny and morphology. Cryptogam. Mycol..

[31] Gafforov Y.S., Abdurazakov A.A., Normakhamatov N.S., Khojimatov O.K., Bussmann R.W. (2021). Uzbekistonning ba’zi dorivor afilloporoid bazidiomiset zamburuglarining etnomikologik ma’lumotlari. Farm. J..

[32] Gafforov Y., Riebesehl J., Ordynets A., Langer E., Yarasheva M., Ghobad-Nejhad M., Zhou L.W., Wang X.W., Gugliota A.M. (2017). *Hyphodontia* (Hymenochaetales, Basidiomycota) and similar taxa from Central Asia. Botany.

[33] Gafforov Y., Ordynets A., Langer E., Yarasheva M., Gugliotta A.M., Schigel D., Pecoraro L., ZhouÌ Y., Cai L., Zhou L.W. (2020). Species diversity with comprehensive annotations of wood inhabiting poroid and corticioid fungi in Uzbekistan. Front. Microbiol..

[34] Antonelli A., Fry C., Smith R.J., Simmonds M.S.J., Kersey P.J., Pritchard H.W., Abbo M.S., Acedo C., Adams J., Ainsworth A.M. (2020). State of the World’s Plants and Fungi 2020.

[35] Cheek M., Lughadha E.N., Kirk P.M., Lindon H.L., Carretero J.M., Looney B.P., Douglas B., Haelewaters D., Haelewaters D., Haelewaters D. (2020). New scientific discoveries: Plants and fungi. Plants People Planet.

[36] Wang X.W., Jiang J.H., Liu S.L., Gafforov Y., Zhou L.W. (2022). Species diversification of the coniferous pathogenic fungal genus *Coniferiporia* (Hymenochaetales, Basidiomycota) in association with its biogeography and host plants. Phytopathology.

[37] Khojimatov O.K., Gafforov Y., Bussmann R.W. (2023). Ethnobiology of Uzbekistan: Ethnomedicinal Knowledge of Mountain Communities.

[38] Gafforov Y., Hoshino T. (2015). Remarks on *Typhula* sp. in Uzbekistan. Mycoscience.

[39] Gafforov Y., Phookamsak R., Jiang H.B., Wanasinghed D.N., Juliev M. (2019). *Ophiobolus hydei* sp. nov. (Phaeosphaeriaceae, Ascomycota) from *Cirsium* and *Phlomoides* in Uzbekistan. Botany.

[40] Yuan Y., Gafforov Y., Chen Y.Y., Wu F. (2017). A new species of *Antrodia* (Basidiomycota, Polyporales) from juniper forest of Uzbekistan. Phytotaxa.

[41] Kan Y.H., Gafforov Y., Li T., Zhou L.W. (2017). *Hyphodontia zhixiangii* sp. nov. (*Schizoporaceae*, Basidiomycota) from Uzbekistan. Phytotaxa.

[42] Appadoo M.A., Wanasinghe D.N., Gafforov Y., Chetana K.T., Abdurazakov A., Hyde K.D., Li Q. (2021). Morphological and phylogenetic insights reveal *Cucurbitaria berberidicola* (*Cucurbitariaceae*, Pleosporales) as a new species from Uzbekistan. Phytotaxa.

[43] Bozorov T.A., Toshmatov Z.O., Kahar G., Zhang D., Shao H., Gafforov Y. (2021). Wild apple-associated fungi and bacteria compete to colonize the larval gut of an invasive wood-borer Agrilus mali in Tianshan Forests. Front. Microbiol..

[44] Aluthmuhandiram J.V.S., Wanasinghe D.N., Chethana K.W.T., Gafforov Y., Saichana N., Li X.H., Yan J., Mamarakhimov O.M. (2022). Lophiostomataceae (Dothideomycetes): Introducing *Lophiostoma khanzadakirgizbaeva* sp. nov. and *Paucispora xishanensis* sp. nov. Phytotaxa.

[45] Zaurov D.E., Belolipov I.V., Kurmukov A.G., Sodombekov I.S., Akimaliev A.A., Eisenman S.W., Eisenman S., Zaurov D., Struwe L. (2013). The Medicinal Plants of Uzbekistan and Kyrgyzstan. Medicinal Plants of Central Asia: Uzbekistan and Kyrgyzstan.

[46] Khojimatov O.K. (2021). Medicinal Plants of Uzbekistan (Properties, Application and Rational Use).

[47] Index Herbariorum. https://sweetgum.nybg.org/science/ih/.

[48] White T.J., Bruns T.D., Lee S., Taylor J., Innis M.A., Gelfand D.H. (1990). Amplification and direct sequencing of fungal ribosomal RNA genes for phylogenetics. PCR Protocols: A Guide to Methods and Applications.

[49] Gardes M., Bruns T.D. (1993). ITS primers with enhanced specificity for basidiomycetes-application to the identification of mycorrhizae and rusts. Mol. Ecol..

[50] http://blast.ncbi.nlm.nih.gov/Blast.cgi.

[51] Katoh K., Standley D.M. (2013). MAFFT multiple sequence alignment software version 7: Improvements in performance and usability. Mol. Biol. Evol..

[52] Katoh K., Kuma K., Toh H., Miyata T. (2005). MAFFT version 5: Improvement in accuracy of multiple sequence alignment. Nucleic Acids Res..

[53] Darriba D., Taboada G.L., Doallo R., Posada D. (2012). jModelTest 2: More models, new heuristics and parallel computing. Nat. Methods.

[54] Guindon S., Gascuel O. (2003). A simple, fast and accurate algorithm to estimate large phylogenies by maximum likelihood. Syst. Biol..

[55] Stamatakis A. (2014). RAxML version 8: A tool for phylogenetic analysis and post-analysis of large phylogenies. Bioinformatics.

[56] Pattengale N.D., Alipour M., Bininda-Emonds O.R.P., Moret B.M.E., Stamatakis A. (2010). How many bootstrap replicates are necessary?. J. Comput. Biol..

[57] Ronquist F., Teslenko M., van der Mark P., Ayres D.L., Darling A., Höhna S., Larget B.R., Liu L., Suchard M.A., Huelsenbeck J.P. (2012). MrBayes 3.2 efficient Bayesian phylogenetic inference and model choice across a large model space. Syst. Biol..

[58] Rambaut A., Drummond A.J., Xie D., Baele G., Suchard M.A. (2018). Posterior summarisation in Bayesian phylogenetics using Tracer 1.7. Syst. Biol..

[59] Xie J.M., Chen Y.R., Cai G.J., Cai R.L., Hu Z., Wang H. (2023). Tree visualization by one table (tvBOT): A web application for visualizing, modifying and annotating phylogenetic trees. Nucleic Acids Res..

[60] Index Fungorum. https://www.indexfungorum.org/.

[61] Plants of the World Online. https://powo.science.kew.org/.

[62] Wu F., Yuan Y., Rivoire B., Dai Y. (2015). Phylogeny and diversity of the *Auricularia mesenterica* (*Auriculariales*, Basidiomycota) complex. Mycol. Prog..

[63] Westphalen M.C., Tomšovský M., Kout J., Gugliotta A.M. (2015). *Bjerkandera* in the Neotropics: Phylogenetic and morphological relations of *Tyromyces atroalbus* and description of a new species. Mycol. Prog..

[64] Floudas D., Hibbett D.S. (2015). Revisiting the taxonomy of Phanerochaete (Polyporales, Basidiomycota) using a four gene dataset and extensive ITS sampling. Fungal Biol..

[65] Badalyan S., Zhuykova E., Mukhin V. (2022). The phylogenetic analysis of Armenian collections of medicinal tinder polypore *Fomes fomentarius* (Agaricomycetes, Polyporaceae). Ital. J. Mycol..

[66] Badalyan S.M., Gharibyan N.G., Iotti M., Zambonelli A. (2019). Morphological and ecological screening of different collections of medicinal white-rot bracket fungus *Ganoderma adspersum* (Schulzer) Donk (*Agaricomycetes*, Polyporales). Ital. J. Mycol..

[67] Zhou L.W., Vlasak J., Vlasak J. (2014). *Inonotus andersonii* and *I. krawtzewii*: Another case of molecular sequencing-based diagnosis of morphologically similar species. Chiang Mai J. Sci..

[68] Vasaitis R., Menkis A., Lim Y.W., Seok S., Tomsovsky M., Jankovsky L., Lygis V., Slippers B., Stenlid J. (2009). Genetic variation and relationships in *Laetiporus sulphureus* s. lat., as determined by ITS rDNA sequences and in vitro growth rate. Mycol. Res..

[69] Ghobad-Nejhad M., Langer E. (2017). First inventory of aphyllophoroid basidiomycetes of Zagros forests, W Iran. Plant Biosyst..

[70] Osmundson T.W., Bergemann S.E., Rasmussen R., Garbelotto M.M. (2022). Using point data to assess biogeographical signal, endemicity and factors associated with macrofungal diversity in the data-poor Pacific oceanic island bioregion. J. Biogeogr..

[71] Ali M., Boekhout T., Thorn R.G. (2017). Assemblages of fungi associated with cork oak forests in northwestern Tunisia. Nova Hedwig..

[72] Hai Bang T., Suhara H., Doi K., Ishikawa H., Fukami K., Parajuli G.P., Katakura Y., Yamashita S., Watanabe K., Adhikari M.K. (2014). Wild mushrooms in Nepal: Some potential candidates as antioxidant and ACE-inhibition sources. Evid. Based Complement. Alternat. Med..

[73] Binder M., Larsson K.H., Matheny P.B., Hibbett D.S. (2010). Amylocorticiales ord. nov. and Jaapiales ord. nov.: Early diverging clades of Agaricomycetidae dominated by corticioid forms. Mykologia.

[74] Binder M., Hibbett D.S. (2006). Molecular systematics and biological diversification of *Boletales*. Mykologia.

[75] He X., Zhao C.L. (2022). Diversity of wood-decaying fungi in Wuliangshan Area, Yunnan Province, P.R. China. Diversity.

[76] Hong Y., Tan J.Y., Xue H., Chow M.L., Ali M., Ng A., Leong A., Yeo J., Koh S.M., Tang M.S.Y. (2023). A metagenomic survey of wood decay fungi in the urban trees of Singapore. J. Fungi.

[77] Gruner O.C. (1930). A Treatise on the Canon of Medicine of Avicenna, Incorporating a Translation of the First Book.

[78] Tayjanov K., Khojimatov O., Gafforov Y., Makhkamov T., Normakhamatov N., Bussmann R.W. (2021). Plants and fungi in the ethnomedicine of the medieval East—A review. Ethnobot. Res. Appl..

[79] Publishing House Fan (1973). Selected Works—Pharmacognosy in Medicine. (Kitāb as-Saidana fi-t-tibb), Herbs and Plants.

[80] Chauhan A.K., Varmat A. (2009). Textbook of Molecular Biotechnology. An Overview.

[81] Lampman A.M. (2007). Ethnomycology: Medicinal and edible mushrooms of the Tzeltal Maya of Chiapas, Mexico. Int. J. Med. Mushrooms.

[82] Wasser S.P., Weis A.L. (1999). Therapeutic effects of substances occurring in higher basidiomycetes mushrooms: A modern perspective. Crit. Rev. Immunol..

[83] Bandara A.R., Rapior S., Mortimer P.E., Kakumyan P., Hyde K.D., Xu J. (2019). A review of the polysaccharide, protein and selected nutrient content of *Auricularia*, and their potential pharmacological value. Mycosphere.

[84] Pak S., Chen F., Ma L., Hu X., Ji J. (2021). Functional perspective of black fungi (*Auricularia auricula*): Major bioactive components, health benefits and potential mechanisms. Trends Food Sci. Technol..

[85] Payamnoor V., Kavosi M., Nazari J. (2020). Polypore fungi of Caucasian alder as a source of antioxidant and antitumor agents. J. For. Res..

[86] Ghosh S., Sett S., Saha R., Roy A., Acharya K. (2021). Comparative phytochemical screening and antioxidant properties of infusion, decoction and hydroalcoholic extracts of wood ear mushrooms; *Auricularia delicata* and *Auricularia mesenterica*. Indian Phytopathol..

[87] Wu F., Tohtirjap A., Fan L.F., Zhou L.W., Alvarenga R.L.M., Gibertoni T.B., Dai Y.C. (2021). Global diversity and updated phylogeny of *Auricularia* (Auriculariales, Basidiomycota). J. Fungi.

[88] Sierra S., Rodríguez-Gutiérrez I., Agustín L.A.I.S., Castro-Santiuste S., Cifuentes J., Pérez-Ramírez L. (2012). Tremelloid fungi (Heterobasidiomycetes) from Calakmul Biosphere Reserve, Campeche, Mexico. Rev. Mex. Biodivers..

[89] Alvarenga R.L.M., Naves L.R.R., Xavier-Santos S. (2015). The genus *Auricularia* Bull. ex Juss. (Basidiomycota) in Cerrado (Brazilian Savanna) areas of Goiás state and the Federal District, Brazil. Mycosphere.

[90] Malysheva V.F., Bulakh E.M. (2014). Contribution to the study of the genus *Auricularia* (Auriculariales, Basidiomycota) in Russia. Nov. Sist. Nizshikh Rastenii.

[91] Rogers R. (2006). The Fungal Pharmacy: Medicinal Mushrooms of Western Canada.

[92] Wu F., Zhou L.W., Yang Z.L., Bau T., Li T.H., Dai Y.C. (2019). Resource diversity of Chinese macrofungi: Edible, medicinal and poisonous species. Fungal Divers..

[93] Chikwem J.O., Jonathan G.S., Hull A., Asemoloye M.D., Osonubi O., Omeonu F.C. (2020). Antimicrobial potential of *Trichaptum biforme* and *Bjerkandera adusta* from Pennsylvania, USA. J. Nat. Sci. Res..

[94] Choo M.J., Hong S.Y., Chung S.H., Om A.S. (2021). Removal of aflatoxin B_1_ by edible mushroom-forming fungi and its mechanism. Toxins.

[95] Ildız E., Canpolat Ş., İşlek C., Yürümez Canpolat E., İşlek Y., Akata I. (2022). *Bjerkandera adusta* collected from Niğde: Analysis of total phenolic compound, antioxidant, and antimicrobial properties. Turk. J. Agric.-Food Sci. Technol..

[96] Ogawa H., Fujimura M., Takeuchi Y., Makimura K. (2009). Is *Bjerkandera adusta* important to fungus-associated chronic cough (FACC) as an allergen? Eight cases’ report. J. Asthma..

[97] Liu B., Ichinose T., He M., Kobayashi F., Maki T., Yoshida S., Yoshida Y., Arashidani K., Takano H., Nishikawa M. (2014). Lung inflammation by fungus, *Bjerkandera adusta* isolated from Asian sand dust (ASD) aerosol and enhancement of ovalbumin-induced lung eosinophilia by ASD and the fungus in mice. Allergy Asthma Clin. Immunol..

[98] Chowdhary A., Agarwal K., Meis J.F. (2016). Filamentous fungi in respiratory infections. What lies beyond aspergillosis and mucormycosis?. PLoS Pathog..

[99] Bernicchia A., Gorjón S.P. (2020). Polypores of the Mediterranean Region.

[100] Zmitrovich I., Bondartseva M., Vasilyev N. (2016). The Meruliaceae of Russia. I. Bjerkandera. Turczaninowia.

[101] Wang C., Vlasák J., Dai Y.C. (2021). Phylogeny and diversity of *Bjerkandera* (Polyporales, Basidiomycota), including four new species from South America and Asia. MycoKeys.

[102] Gafforov Y., Ordynets A. (2022). Aphyllophoroid Fungi of Uzbekistan.

[103] Babakhin A., Logina N.Y., Nolte H., Dubuske L.M. (1996). Immunomodulating activity of the extract from high mycelium fungus *Polyporus squamosus*. J. Allergy Clin. Immunol..

[104] Babakhin A., Vedernikov A.A., Babakhin A.A., Vp L., Vm P., Logina N.Y., Guschin L.S., Holte H., Dubuske L.M. (1999). Immunosuppressive activity of the extract of high mycelium fungus *Polyporus squamosus*. Immunologiya.

[105] Elmastas M., Isildak O., Turkekul I., Temur N. (2007). Determination of antioxidant activity and antioxidant compounds in wild edible mushrooms. J. Food Compost. Anal..

[106] Ferreira I.C., Barros L., Abreu R.M. (2009). Antioxidants in wild mushrooms. Curr. Med. Chem..

[107] Zhao Y.Y., Xie R.M., Chao X., Zhang Y., Lin R.C., Sun W. (2009). Bioactivity-directed isolation, identification of diuretic compounds from *Polyporus umbellatus*. J. Ethnopharmacol..

[108] Dimitrijevic M., Stankov Jovanovic V., Cvetkovic J., Mihajilov-Krstev T., Stojanovic G., Mitic V. (2015). Screening of antioxidant, antimicrobial and antiradical activities of twelve selected Serbian wild mushrooms. Anal. Methods..

[109] Meunink J. (2015). Basic Illustrated Edible and Medicinal Mushrooms.

[110] Fernandes Â., Petrović J., Stojković D., Barros L., Glamočlija J., Soković M.D., Martins A., Ferreira I.C. (2016). *Polyporus squamosus* (Huds.) Fr from different origins: Chemical characterization, screening of the bioactive properties and specific antimicrobial effects against *Pseudomonas aeruginosa*. LWT—Food Sci. Technol..

[111] Elkhateeb W.A., Daba G.M., Elnahas M.O., Thomas P.W., Emam M. (2020). Metabolic profile and skin-related bioactivities of *Cerioporus squamosus* hydromethanolic extract. Biodiversitas.

[112] Liu B. (1984). The Chinese Medical Fungi.

[113] Ying J., Mao X., Ma Q., Zong Y., Wen H. (1987). Icones of Medical Fungi from China.

[114] Demir M.S., Yamac M. (2008). Antimicrobial activities of basidiocarp, submerged mycelium and exopolysaccharide of some native Basidiomycetes strains. J. Appl. Biol. Sci..

[115] Yamac M., Zeytinoglu M., Kanbak G., Bayramoglu G., Senturk H. (2009). Hypoglycemic effect of crude exopolysaccharides produced by *Cerrena unicolor*, *Coprinus comatus* and *Lenzites betulina* isolates in streptozotocin-induced diabetic rats. Pharm. Biol..

[116] Uyanoglu M., Canbek M., Ozalp F.O., Yamac M., Senturk H. (2011). Effects of some macrofungi exopolysaccharides on mesenchymal mast cells of rats in chronic alcohol consumption. Int. J. Health Nutr..

[117] Jaszek M., Osińska-Jaroszuk M., Janusz G., Matuszewska A., Stefaniuk D., Sulej J., Polak J., Ruminowicz M., Grzywnowicz K., Jarosz-Wilkolazka A. (2013). New bioactive fungal molecules with high antioxidant and antimicrobial capacity isolated from *Cerrena unicolor* idiophasic cultures. BioMed Res. Int..

[118] Mizerska-Dudka M., Jaszek M., Blachowicz A., Rejczak T.P., Matuszewska A., Osińska-Jaroszuk M., Stefaniuk D., Janusz G., Sulej J., Kandefer-Szerszeń M. (2015). Fungus *Cerrena unicolor* as an effective source of new antiviral, immunomodulatory, and anticancer compounds. Int. J. Biol. Macromol..

[119] Statkiewicz M., Matuszewska A., Jaszek M., Janusz G., Osińska M., Sulej J., Stefaniuk D., Mikula M., Ostrowski J. (2017). Antimelanomic effects of high- and low-molecular weight bioactive subfractions isolated from the mossy maze mushroom, *Cerrena unicolor* (Agaricomycetes). Int. J. Med. Mushrooms.

[120] Matuszewska A., Jaszek M., Stefaniuk D., Ciszewski T., Matuszewski Ł. (2018). Anticancer, antioxidant, and antibacterial activities of low molecular weight bioactive subfractions isolated from cultures of wood degrading fungus *Cerrena unicolor*. PLoS ONE.

[121] Matuszewska A., Stefaniuk D., Jaszek M., Pięt M., Zając A., Matuszewski Ł., Cios I., Grąz M., Paduch R., Bancerz R. (2019). Antitumor potential of new low molecular weight antioxidative preparations from the white rot fungus *Cerrena unicolor* against human colon cancer cells. Sci. Rep..

[122] Stefaniuk D., Misztal T., Piet M., Zajac A., Kopycinska M., Matuszewska A., Ruminowicz-Stefaniuk M., Matuszewski Ł., Marcinczyk N., Belcarz A. (2021). Thromboelastometric analysis of anticancer *Cerrena unicolor* subfractions reveal their potential as fibrin glue drug carrier enhancers. Biomolecules.

[123] Roussel B., Rapior S. (2005). Les Usages de l’Amadouvier en Odontologie. Ann. Soc. Hortic. Hist. Nat. Hérault.

[124] Karaman M., Jovin E., Malbaša R., Matavuly M., Popović M. (2010). Medicinal and edible lignicolous fungi as natural sources of antioxidative and antibacterial agents. Phytother. Res..

[125] Patel S., Goyal A. (2012). Recent developments in mushrooms as anti-cancer therapeutics: A review. 3 Biotech..

[126] Vazirian M., Dianat S., Manayi A., Ziari R., Mousazadeh A., Habibi E., Saeidnia S., Amanzadeh Y. (2014). Anti-inflammatory effect, total polysaccharide, total phenolics content and antioxidant activity of the aqueous extract of three basidiomycetes. Res. J. Pharmacogn..

[127] Kim S.H., Jakhar R., Kang S.C. (2015). Apoptotic properties of polysaccharide isolated from fruiting bodies of medicinal mushroom *Fomes fomentarius* in human lung carcinoma cell line. Saudi J. Biol. Sci..

[128] Kolundžić M., Grozdanić N.D., Dodevska M., Milenković M., Sisto F., Miani A., Farronato G., Kundaković T. (2016). Antibacterial and cytotoxic activities of wild mushroom *Fomes fomentarius* (L.) Fr., Polyporaceae. Ind. Crops Prod..

[129] Maljurić N., Golubović J., Ravnikar M., Žigon D., Borut Š., Otašević B. (2018). Isolation and determination of fomentariol: Novel potential antidiabetic drug from fungal material. J. Anal. Method. Chem..

[130] Deveci E., Çayan F., Tel-Çayan G., Duru M.E. (2019). Structural characterization and determination of biological activities for different polysaccharides extracted from tree mushroom species. J. Food Biochem..

[131] Gedik G., Dulger G., Asan H., Ozyurt A., Alli H., Asan A. (2019). The antimicrobial effect of various formulations obtained from *Fomes fomentarius* against hospital isolates. Mantar Derg..

[132] Lee S.O., Lee M.H., Lee K.R., Lee E.O., Lee H.J. (2019). *Fomes fomentarius* ethanol extract exerts inhibition of cell growth and motility induction of apoptosis via targeting AKT in human breast cancer MDA-MB-231 cells. Int. J. Mol. Sci..

[133] Alvandi H., Hatamian-Zarmi A., Ebrahimi Hosseinzadeh B., Mokhtari-Hosseini Z.B. (2020). Optimization of production conditions for bioactive polysaccharides from *Fomes fomentarius* and investigation of antibacterial and antitumor activities. Iran. J. Med. Microbiol..

[134] Storsberg J., Krüger-Genge A., Kalitukha L. (2022). In vitro cytotoxic activity of an aqueous alkali extract of the tinder conk mushroom, *Fomes fomentarius* (Agaricomycetes), on murine fibroblasts, human colorectal adenocarcinoma, and cutaneous melanoma cells. Int. J. Med. Mushrooms.

[135] Kalitukha L., Galiano A., Harrison F. (2023). Medicinal potential of the insoluble extracted fibers isolated from the *Fomes fomentarius* (Agaricomycetes) fruiting bodies: A review. Int. J. Med. Mushrooms.

[136] Stamets P. (1993). Growing Gourmet and Medicinal Mushrooms.

[137] Peintner U., Kuhnert-Finkernagel R., Wille V., Biasioli F., Shiryaev A., Perini C. (2019). How to resolve cryptic species of polypores: An example in *Fomes*. IMA Fungus.

[138] Hobbs C. (2002). Medicinal Mushrooms: An Exploration of Tradition, Healing, and Culture.

[139] Rutalek R. (2002). Ethnomykologie–Eine Übersicht. Osterr. Z. Pilzkd..

[140] Lemieszek M.K., Langner E., Kaczor J., Kandefer-Szerszen M., Sanecka B., Mazurkiewicz W., Rzeski W. (2009). Anticancer effect of fraction isolated from medicinal birch polypore mushroom, *Piptoporus betulinus* (Bull.: Fr.) P. Karst. (Aphyllophoromycetideae): In vitro studies. Int. J. Med. Mushrooms.

[141] Grienke U., Zöll M., Peintner U., Rollinger J.M. (2014). European medicinal polypores—A modern view on traditional uses. J. Ethnopharmacol..

[142] Papp N., Rudolf K., Bencsik T., Czégényi D. (2017). Ethnomycological use of *Fomes fomentarius* (L.) Fr. and *Piptoporus betulinus* (Bull.) P. Karst. in Transylvania, Romania. Genet. Resour. Crop Evol..

[143] Krupodorova T.A., Barshteyn V.Y., Pokas E.V. (2019). Antibacterial activity of *Fomitopsis betulina* cultural liquid. EUREKA: Life Sci..

[144] Yong-Tae J., Yang B.K., Jeong S.C., Kim S.M., Song C.H. (2008). *Ganoderma applanatum*: A promising mushroom for antitumor and immunomodulating activity. Phytother. Res..

[145] Kinge T.R., Tabi E.M., Mih A.M., Enow E.A., Njouonkou L., Nji T.M. (2011). Ethnomycological studies of edible and medicinal mushrooms in the Mount Cameroon region (Cameroon, Africa). Int. J. Med. Mushrooms.

[146] Rašeta M., Popović M., Beara I., Šibul F., Zengin G., Krstić S., Karaman M. (2020). Anti-inflammatory, antioxidant and enzyme inhibition activities in correlation with mycochemical profile of selected indigenous *Ganoderma* spp. from Balkan region (Serbia). Chem. Biodivers..

[147] Mfopa A., Mediesse F.K., Mvongo C., Nkoubatchoundjwen S., Lum A.A., Sobngwi E., Kamgang R., Boudjeko T. (2021). Antidyslipidemic potential of water-soluble polysaccharides of *Ganoderma applanatum* in MACAPOS-2-induced obese rats. Evid. Based Complement. Altern. Med..

[148] Sułkowska-Ziaja K., Zengin G., Gunia-Krzyżak A., Popiół J., Szewczyk A., Jaszek M., Rogalski J., Muszyńska B. (2022). Bioactivity and mycochemical profile of extracts from mycelial cultures of *Ganoderma* spp.. Molecules.

[149] Sadava D., Still D.W., Mudry R.R., Kane S.E. (2009). Effect of *Ganoderma* on drug-sensitive and multidrug resistant small-cell lung carcinoma cells. Cancer Lett..

[150] De Silva D.D., Rapior S., Fons F., Bahkali A.H., Hyde K.D. (2012). Medicinal mushrooms in supportive cancer therapies: An approach to anti-cancer effects and putative mechanisms of action. Fungal Divers..

[151] De Silva D.D., Rapior S., Hyde K.D., Bahkali A.H. (2012). Medicinal mushrooms in prevention and control of diabetes mellitus. Fungal Divers..

[152] Tel-Çayan G., Öztürk M., Duru M.E., Rehman M.U., Adhikari A., Türkoğlu A., Choudhary M.I. (2015). Phytochemical investigation, antioxidant and anticholinesterase activities of *Ganoderma adspersum*. Ind. Crops Prod..

[153] Tel-Çayan G., Muhammad A., Deveci E., Duru M.E., Öztürk M. (2020). Isolation, structural characterization, and biological activities of galactomannans from *Rhizopogon luteolus* and *Ganoderma adspersum* mushrooms. Int. J. Biol. Macromol..

[154] Hapuarachchi K.K., Cheng C.R., Wen T.C., Jeewon R., Kakumyan P. (2017). Mycosphere Essays 20: Therapeutic potential of *Ganoderma* species: Insights into its use as traditional medicine. Mycosphere.

[155] Mayaka R.K., Njue A.W., Langat M.K., Cheplogoi P.K., Omolo J.O. (2020). Antimicrobial compounds from the Kenyan *Ganoderma adspersum* (Schulz.) Donk species. Int. J. Biol. Chem. Sci..

[156] Xiao C., Wu Q., Xie Y., Zhang J., Tan J. (2015). Hypoglycemic effects of *Grifola frondosa* (Maitake) polysaccharides F2 and F3 through improvement of insulin resistance in diabetic rats. Food Funct..

[157] Bao H., Ran P., Sun L., Hu W., Li H., Xiao C., Zhu K., Du J. (2016). *Grifola frondosa* (GF) produces significant antidepressant effects involving AMPA receptor activation in mice. Pharm. Biol..

[158] Bai Y., Chen L., Chen Y., Chen X., Dong Y., Zheng S., Zhang L., Li W., Du J., Li H. (2019). A Maitake (*Grifola frondosa*) polysaccharide ameliorates Alzheimer’s disease-like pathology and cognitive impairments by enhancing microglial amyloid-β clearance. RSC Adv..

[159] Gargano M.L., Zervakis G.I., Isikhuemhen O.S., Venturella G., Calvo R., Giammanco A., Fasciana T., Ferraro V. (2020). Ecology, phylogeny, and potential nutritional and medicinal value of a rare white “Maitake” collected in a Mediterranean forest. Diversity.

[160] Hetland G., Tangen J., Mahmood F., Mirlashari M.R., Nissen-Meyer L.S., Nentwich I., Therkelsen S.P., Tjønnfjord G.E., Johnson E. (2020). Antitumor, anti-inflammatory and antiallergic effects of *Agaricus blazei* mushroom extract and the related medicinal Basidiomycetes mushrooms, *Hericium erinaceus* and *Grifola frondosa*: A review of preclinical and clinical studies. Nutrients.

[161] Jiang T., Wang L., Ma A., Wu Y., Wu Q., Wu Q., Lu J., Zhong T. (2020). The hypoglycemic and renal protective effects of *Grifola frondosa* polysaccharides in early diabetic nephropathy. J. Food Biochem..

[162] Wu S.J., Tung Y.J., Ng L.T. (2020). Anti-diabetic effects of *Grifola frondosa* bioactive compound and its related molecular signaling pathways in palmitate-induced C_2_C_12_ cells. J. Ethnopharmacol..

[163] Wu J.Y., Siu K.C., Geng P. (2021). Bioactive ingredients and medicinal values of *Grifola frondosa* (Maitake). Foods.

[164] Aranaz P., Peña A.D., Vettorazzi A., Fabra M.J., Martínez-Abad A., López-Rubio A., Pera J., Parladé J., Castellari M., Milagro F.I. (2021). *Grifola frondosa* (Maitake) extract reduces fat accumulation and improves health span in *C. elegans* through the DAF-16/FOXO and SKN-1/NRF2 signalling pathways. Nutrients.

[165] Fasciana T., Gargano M.L., Serra N., Galia E., Arrigo I., Tricoli M.R., Diquattro O., Graceffa G., Vieni S., Venturella G. (2021). Potential activity of Albino *Grifola frondosa* mushroom extract against biofilm of meticillin-resistant *Staphylococcus aureus*. J. Fungi.

[166] Wang C., Zeng F., Liu Y., Pan Y., Xu J., Ge X., Zheng H., Pang J., Liu B., Huang Y. (2021). Coumarin-rich *Grifola frondosa* ethanol extract alleviate lipid metabolism disorders and modulates intestinal flora compositions of high-fat diet rats. J. Funct. Foods.

[167] Yousfi M., Djeridane A., Bombarda I., Chahrazed-Hamia Duhem B., Gaydou E.M. (2009). Isolation and characterization of a new hispolone derivative from antioxidant extracts of *Pistacia atlantica*. Phytother. Res..

[168] Alves M.J., Ferreira I.C., Dias J., Teixeira V., Martins A., Pintado M.A. (2012). Review on antimicrobial activity of mushroom (Basidiomycetes) extracts and isolated compounds. Planta Med..

[169] Dugger B.N., Dickson D.W. (2016). Pathology of neurodegenerative diseases. Cold Spring Harb. Perspect. Biol..

[170] Ren Q., Lu X., Han J., Aisa H.A., Yuan T. (2017). Triterpenoids and phenolics from the fruiting bodies of *Inonotus hispidus* and their activations of melanogenesis and tyrosinase. Chin. Chem. Lett..

[171] Angelini P., Girometta C., Tirillini B., Moretti S., Covino S., Cipriani M., D’Ellena T., Angeles G., Federici E., Savino E. (2019). A comparative study of the antimicrobial and antioxidant activities of *Inonotus hispidus* fruit and their mycelia extracts. Int. J. Food Prop..

[172] Kou R.W., Du S.T., Xia B., Zhang Q., Yin X., Gao J.M. (2021). Phenolic and steroidal metabolites from the cultivated edible *Inonotus hispidus* mushroom and their bioactivities. J. Agric. Food Chem..

[173] Zhang Y., Hao J., Liu Z., Li Z., Teng L., Wang D. (2022). *Inonotus hispidus* protects against hyperlipidemia by inhibiting oxidative stress and inflammation through Nrf2/NF-κB signaling in high fat diet fed mice. Nutrients.

[174] Sułkowska-Ziaja K., Muszyńska B., Gawalska A., Sałaciak K. (2018). *Laetiporus sulphureus*—Chemical composition and medicinal value. Acta Sci. Pol. Hortorun Cultus.

[175] Adamska I. (2023). The possibility of using sulphur shelf fungus (*Laetiporus sulphureus*) in the food industry and in medicine-a review. Foods.

[176] Dulay R.M., Arenas M.C., Kalaw S.P., Reyes R.G., Cabrera E.C. (2014). Proximate composition and functionality of the culinary-medicinal tiger sawgill mushroom, *Lentinus tigrinus* (higher Basidiomycetes), from the Philippines. Int. J. Med. Mushrooms.

[177] Malik A.R., Wani A.H., Bhat M.Y., Parveen S. (2017). Ethnomycologicl knowledge of some wild mushrooms of northern districts of Jammu and Kashmir, India. Asian J. Pharm. Clin. Res..

[178] Ragasa C., Tan M., De Castro M.E., De Los Reyes M., Oyong G., Shen C.C. (2018). Sterols from *Lentinus tigrinus*. Pharmacogn. J..

[179] Sevindik M. (2018). Investigation of antioxidant/oxidant status and antimicrobial activities of *Lentinus tigrinus*. Adv. Pharmacol. Sci..

[180] Gao Y., Wáng Y., Wang Y., Wu Y., Chen H., Yang R.H., Bao D. (2019). Protective function of novel fungal immunomodulatory proteins Fip-lti1 and Fip-lti2 from *Lentinus tigrinus* in concanavalin a-induced liver oxidative injury. Oxid. Med. Cell. Longev..

[181] Mohammadnejad S., Pourianfar H., Drakhshan A., Jabaleh I., Rezayi M. (2019). Potent antiproliferative and pro-apoptotic effects of a soluble protein fraction from culinary-medicinal mushroom *Lentinus tigrinus* on cancer cells. J. Food Meas. Charact..

[182] Pourianfar H.R., Mohammadnejad S., Shahtahmasebi S., Ansari A.M., Zibaei S., Ghadirian R., Rezaeian S., Dowom S.A. (2020). Toxicity and nutritional assessment of extracts of medicinal Tiger Sawgill mushroom, *Lentinus tigrinus* (Agaricomycetes), a newly domesticated in Iran. Int. J. Med. Mushrooms.

[183] Torres M.L., Reyes R., Tadiosa E., Ontengco D. (2020). Ethnomycological studies on the Bugkalot Indigenous Community in Alfonso Castañeda, Nueva Vizcaya, Philippines. Int. J. Pharm. Res. Allied Sci..

[184] Blanchette R.A., Renner C.C., Held B.W., Enoch C., Angstman S. (2002). The current use of *Phellinus igniarius* by the Eskimos of western Alaska. Mycology.

[185] Wang J.Z., Wu L., Luo Y.C., Zhang H.Q., Huang W.F., Yan X.M., Zhang P. (2015). Effects of ethanol extracts of *Phellinus lonicerinus* on hepatic stellate cells of fibrosis liver in rats. Zhong Yao Cai.

[186] Wang J., Lv H., Chen B., Huang W., Wang A., Liu L., He H., Chen J., Li S., Deng W. (2019). Methyl-hispolon from *Phellinus lonicerinus* (Agaricomycetes) affects estrogen signals in MCF-7 breast cancer cells and premature aging in rats. Int. J. Med. Mushrooms.

[187] Badalyan S.M., Gharibyan N.G. (2020). Pharmacological properties and resource value of Hymenochaetoid fungi (Agaricomycetes) distributed in Armenia: Review. Int. J. Med. Mushrooms.

[188] He P., Zhang Y., Li N. (2021). The phytochemistry and pharmacology of medicinal fungi of the genus *Phellinus*: A review. Food Funct..

[189] Li Y., Zhang Z., Feng Y., Cheng Y., Li S., Li C., Tian L. (2021). Cardioprotective 22-hydroxylanostane triterpenoids from the fruiting bodies of *Phellinus igniarius*. Phytochemistry.

[190] Wu X., Lin S., Zhu C., Zhao F., Yu Y., Yue Z., Liu B., Yang Y., Dai J., Shi J. (2011). Studies on constituents of cultures of fungus *Phellinus igniarius*. Zhongguo Zhong Yao Za Zhi.

[191] Zhou L.W., Ghobad-Nejhad M., Tian X.M., Wang Y.F., Wu F. (2022). Current status of ‘Sanghuang’ as a group of medicinal mushrooms and their perspective in industry development. Food Rev. Int..

[192] Hobbs C.R. (2005). The chemistry, nutritional value, immunopharmacology, and safety of the traditional food of medicinal split-gill fugus *Schizophyllum commune* Fr.:Fr. (*Schizophyllaceae*). A literature review. Int. J. Med. Mushrooms.

[193] Devi L.S., Dasgupta A., Chakraborty M., Borthakur S.K., Singh N.I. (2014). Chemical composition and antioxidant activity of *Schizophyllum commune*. Int. J. Pharm. Sci. Rev. Res..

[194] Du B., Zeng H.S., Yang Y.D., Bian Z.X., Xu B.J. (2016). Anti-inflammatory activity of polysaccharide from *Schizophyllum commune* as affected by ultrasonication. Int. J. Biol. Macromol..

[195] Yao H.M., Wang G., Liu Y.P., Rong M.Q., Shen C.B., Yan X.W., Luo X., Lai X.D. (2016). Phenolic acids isolated from the fungus *Schizophyllum commune* exert analgesic activity by inhibiting voltage-gated sodium channels. Chin. J. Nat. Med..

[196] Abd Razak D.L., Jamaluddin A., Rashid N.Y., Fadzil N.H.M., Sani N.A., Manan M.A. (2018). Comparative evaluation of *Schizophyllum commune* extracts as potential cosmeceutical bio-ingredient. Int. J. Res. Agric. Sci..

[197] Chen Z.Y., Yin C.M., Fan X.Z., Ma K., Yao F., Zhou R.R., Shi D.F., Cheng W., Gao H. (2020). Characterization of physicochemical and biological properties of *Schizophyllum commune* polysaccharide extracted with different methods. Int. J. Biol. Macromol..

[198] Khardziani T., Metreveli E., Didebulidze K., Elisashvili V. (2020). Screening of Georgian medicinal mushrooms for their antibacterial activity and optimization of cultivation conditions for the split gill medicinal mushroom, *Schizophyllum commune* BCC64 (Agaricomycetes). Int. J. Med. Mushrooms.

[199] Mišković J., Karaman M., Rašeta M., Krsmanović N., Berežni S., Jakovljević D.V., Piattoni F., Zambonelli A., Gargano M.L., Venturella G. (2021). Comparison of two *Schizophyllum commune* strains in production of acetylcholinesterase inhibitors and antioxidants from submerged cultivation. J. Fungi.

[200] Mišković J., Rašeta M., Krsmanović N., Karaman M. (2023). Update on mycochemical profile and selected biological activities of genus *Schizophyllum* Fr. 1815. Microbiol. Res..

[201] Vu V., Muthuramalingam K., Singh V., Choi C., Kim Y.M., Unno T., Cho M. (2022). *Schizophyllum commune*-derived β-glucan improves intestinal health demonstrating protective effects against constipation and common metabolic disorders. Appl. Biol. Chem..

[202] Zeynali M., Alvandi H., Hatamian Zarmi A., Yasrebi N., Mokhtari-Hosseini Z.B., Mohammadi M., Larypoor M. (2022). *Schizophyllum commune*-derived chitin glucan complex wound dressing: Antibacterial activity and wound healing properties in a second degree burn animal model. J. Nat. Fibers.

[203] Kumar M., Harsh N.S.K., Prasad R., Pandey V.V. (2017). An ethnomycological survey of Jaunsar, Chakrata, Dehradun. J. Threat. Taxa..

[204] Duan Y., Feng J., Bai N.S., Li G., Zhang K., Zhao P. (2018). Four novel antibacterial sesquiterpene-α-amino acid quaternary ammonium hybrids from the mycelium of mushroom *Stereum hirsutum*. Fitoterapia.

[205] Hu Q., Duan Y., Pu X., Li S., Li G., Zhao P. (2020). Stereumamides E-H, four new minor quaternary ammonium hybrids from *Stereum hirsutum*. Nat. Prod. Res..

[206] Tian M., Zhao J., Li G., Zhang K. (2020). In depth natural product discovery from the Basidiomycetes *Stereum* species. Microorganisms.

[207] Mišković J., Rašeta M., Čapelja E., Krsmanović N., Novaković A., Janjušević L., Karaman M. (2021). Mushroom species *Stereum hirsutum* as natural source of phenolics and fatty acids as antioxidants and acetylcholinesterase inhibitors. Chem. Biodivers..

[208] Zhao Z., Zhao X., Si Y., Wang Z., Sun Y., Chen H., Feng W., Liu J. (2022). Structure elucidation of linear triquinane sesquiterpenoids, hirsutuminoids A-Q, from the fungus *Stereum hirsutum* and their activities. Phytochemistry.

[209] Chu K., Ho S.S., Chow A.H. (2002). *Coriolus versicolor*: A medicinal mushroom with promising immunotherapeutic values. J. Clin. Pharmacol..

[210] Chang Y., Zhang M., Jiang Y., Liu Y., Luo H., Hao C., Zeng P., Zhang L. (2017). Preclinical and clinical studies of *Coriolus versicolor* polysaccharopeptide as an immunotherapeutic in China. Discov. Med..

[211] Dou H., Chang Y., Zhang L. (2019). *Coriolus versicolor* polysaccharopeptide as an immunotherapeutic in China. Prog. Mol. Biol. Transl. Sci..

[212] Rašeta M., Popović M., Knežević P., Šibul F., Kaišarević S., Karaman M. (2020). Bioactive phenolic compounds of two medicinal mushroom species *Trametes versicolor* and *Stereum subtomentosum* as antioxidant and antiproliferative agents. Chem. Biodivers..

[213] Krsmanović N., Rašeta M., Mišković J., Bekvalac K., Bogavac M., Karaman M., Isikhuemhen O.S. (2023). Effects of UV stress in promoting antioxidant activities in fungal species *Trametes versicolor* (L.) Lloyd and *Flammulina velutipes* (Curtis) Singer. Antioxidants.

[214] Ryvarden L., Gilbertson R.L. (1994). European Polypores. Part 2. Synopsis Fungorum.

[215] Ryvarden L., Melo I. (2014). Poroid Fungi of Europe. Synopsis Fungorum 31.

[216] Gründemann C., Reinhardt J.K., Lindequist U. (2020). European medicinal mushrooms: Do they have potential for modern medicine?—An update. Phytomedicine.

[217] Band Z., Frerichs G., Arends G., Zörnig H. (2013). Hagers Handbuch der Pharmazeutischen Praxis: Für Apotheker, Arzneimittelhersteller, Drogisten, Ärzte und Medizinalbeamte.

[218] Peintner U., Pöder R., Bortenschlager S., Oeggl K. (2000). Ethnomycological remarks on the Iceman’s fungi. The Iceman and His Natural Environment.

[219] Roussel B., Rapior S., Masson C.L., Boutié P., Mimosa (2002). L’Amadouvier. Grande et Petite Histoire d’un Champignon.

[220] Ryvarden L., Gilbertson R.L. (1993). European Polypores. Part 1.

[221] Vunduk J., Klaus A., Kozarski M., Petrović P.M., Zizak Z., Nikšić M.P., Van Griensven L.J. (2015). Did the Iceman know better? Screening of the medicinal properties of the birch Polypore medicinal mushroom, *Piptoporus betulinus* (Higher Basidiomycetes). Int. J. Med. Mushrooms.

[222] Shamtsyan M., Konusova V., Maksimova Y., Goloshchev A., Panchenko A., Simbirtsev A., Petrishchev N., Denisova N. (2004). Immunostimulating and anti-tumor action of extracts of several mushrooms. J. Biotechnol..

[223] Chang S.T., Miles P.G. (2004). Mushrooms: Cultivation, Nutritional Value, Medicinal Effect, and Environmental Impact.

[224] Gilbertson R.L., Ryvarden L. (1987). North American Polypores.

[225] Dai Y.C., Yang Z.L., Cui B.K., Yu C.Y., Zhou Z.W. (2009). Species diversity and utulization of medicinal mushrooms and fungi in China. Int. J. Med. Mushrooms.

[226] Oyetayo O.V. (2011). Medicinal uses of mushrooms in Nigeria: Towards full and sustainable exploitation. Afr. J. Tradit. Complement. Altern. Med..

[227] Pala S.A., Wani A.H., Bhat M.Y. (2013). Ethnomycological studies of some wild medicinal and edible mushrooms in the Kashmir Himalayas (India). Int. J. Med. Mushrooms.

[228] Kumaran S., Pandurangan A.K., Shenbhagaraman R., Esa N.M. (2017). Isolation and characterization of lectin from the Artist’s Conk medicinal mushroom, *Ganoderma applanatum* (Agaricomycetes), and evaluation of its antiproliferative activity in HT-29 colon cancer cells. Int. J. Med. Mushrooms.

[229] Peng X.R., Wang Q., Su H.G., Zhou L., Xiong W.Y., Qiu M.H. (2022). Anti-adipogenic lanostane-type triterpenoids from the edible and medicinal mushroom *Ganoderma applanatum*. J. Fungi.

[230] Beck T., Gáperová S., Gáper J., Náplavová K., Šebesta M., Kisková J., Pristaš P. (2020). Genetic (non)-homogeneity of the bracket fungi of the genus *Ganoderma* (Basidiomycota) in Central Europe. Mycosphere.

[231] Shin Y., Lee S. (2014). Antioxidant activity and β-glucan contents of hydrothermal extracts from maitake (*Grifola frondosa*). Food Sci. Biotechnol..

[232] Acharya K., Bera I., Khatua S., Rai M. (2015). Pharmacognostic standardization of *Grifola frondosa*: A well-studied medicinal mushroom. Pharm. Lett..

[233] Konno S., Alexander B., Zade J., Choudhury M. (2013). Possible hypoglycemic action of SX-fraction targeting insulin signal transduction pathway. Int. J. Gen. Med..

[234] Patel D.K., Seo Y., Dutta S.D., Lee O., Lim K. (2020). Influence of Maitake (*Grifola frondosa*) particle sizes on human mesenchymal stem cells and in vivo evaluation of their therapeutic potential. Biomed. Res. Int..

[235] Gu C., Li J., Chao F., Jin M., Wang X., Shen Z. (2007). Isolation, identification and function of a novel anti-HSV-1 protein from *Grifola frondosa*. Antivir. Res..

[236] Sato M., Tokuji Y., Yoneyama S., Fujii-Akiyama K., Kinoshita M., Chiji H., Ohnishi M. (2013). Effect of dietary Maitake (*Grifola frondosa*) mushrooms on plasma cholesterol and hepatic gene expression in cholesterol-fed mice. J. Oleo Sci..

[237] Zhao S., Gao Q., Rong C., Wang S., Zhao Z., Liu Y., Xu J. (2020). Immunomodulatory effects of edible and medicinal mushrooms and their bioactive immunoregulatory products. J. Fungi.

[238] Shevchenko M.V., Heluta V.P., Hayova V.O. (2019). Distribution and conservation status of *Grifola frondosa* (Polyporales, Basidiomycota) in Ukraine. Ukr. Bot. J..

[239] Davoli P., Mucci A., Schenetti L., Weber R.W.S. (2005). Laetiporic acids, a family of non-carotenoid polyene pigments from fruit-bodies and liquid cultures of *Laetiporus sulphureus* (Polyporales, Fungi). Phytochemistry.

[240] Elkhateeb W.A., El Ghwas D.E., Gundoju N.R., Somasekhar T., Akram M., Daba G.M. (2021). Chicken of the woods *Laetiporus sulphureus* and *Schizophyllum commune* treasure of medicinal mushrooms. J. Microbiol. Biotechnol..

[241] Bulam S., Üstün N., Peksen A. (2019). Nutraceutical and food preserving importance of *Laetiporus sulphureus*. Turk. J. Agric.-Food Sci. Technol..

[242] Breitenbach J., Kränzlin F. (1991). Fungi of Switzerland.

[243] Shen S., Liu S.L., Jiang J.H., Zhou L.W. (2021). Addressing widespread misidentifications of traditional medicinal mushrooms in Sanghuangporus (Basidiomycota) through ITS barcoding and designation of reference sequences. IMA Fungus.

[244] Parmasto E., Parmasto I. (2001). *Phellinus baumii* and related species of the *Ph. linteus* group (Hymenochaetaceae, Hymenomycetes). Folia Cryptogam. Est..

[245] Cappello García S., Carreño Ruiz S.D., Gaitán Hernández R., Sánchez J.E., Mata G., Royse D.J. (2018). Fruit body production of *Schizophyllum commune*. Updates on Tropical Mushrooms. Basic and Applied Research.

[246] Milenge Kamalebo H., Nshimba Seya wa Malale H., Masumbuko Ndabaga C., Degreef J., De Kesel A. (2018). Uses and importance of wild fungi: Traditional knowledge from the Tshopo province in the Democratic Republic of the Congo. J. Ethnobiol. Ethnomed..

[247] Sharma A., Kaur R., Kaur J., Garg S., Bhatti R., Kaur A. (2021). An endophytic *Schizophyllum commune* Fr. exhibits in vitro and in vivo antidiabetic activity in streptozotocin induced diabetic rats. AMB Express.

[248] Tripathi A., Tiwary B.N. (2013). Biochemical constituents of a wild strain of *Schizophyllum commune* isolated from Achanakmar-Amarkantak Biosphere Reserve (ABR), India. World J. Microb. Biot..

[249] Umeo S.H., Faria M.G., Dragunski D.C., Valle J.S., Colauto N.B., Linde G.A. (2020). Iron or zinc bioaccumulated in mycelial biomass of edible Basidiomycetes. Ann. Acad. Bras. Cienc..

[250] Garcia J., Rodrigues F., Saavedra M.J., Nunes F.M., Marques G. (2022). Bioactive polysaccharides from medicinal mushrooms: A review on their isolation, structural characteristics and antitumor activity. Food Biosci..

[251] Chen X., Sun J., Chen Y., Wang J., Liu S. (2023). Pneumonia caused by *Schizophyllum commune* in a patient with diabetes: A case report and comprehensive literature review. Medicine.

[252] Zhang J., Xu G., Shen J., Ye G. (2023). Pulmonary infection of *Schizophyllum commune* diagnosed by metagenomic next-generation sequencing: A case report. Medicine.

[253] Ryvarden L. (2020). The genus *Stereum*—A synopsis. Synop. Fungorum.

[254] Ming A. Chinese-English Manual of Common Used in Traditional Chinese Medicine; Publishing House of Guangdong Science and Technology, Guangzhou, Guangdong Province, China: 1989. mycomedica.eu.

[255] Li F., Wen H., Zhang Y., Aa M., Liu X. (2011). Purification and characterization of a novel immunomodulatory protein from the medicinal mushroom *Trametes versicolor*. Sci. China Life Sci..

[256] Razmovski-Naumovski V., Kimble B., Laurenti D., Nammi S., Norimoto H., Chan K. (2022). Polysaccharide peptide extract from *Coriolus versicolor* increased tmax of tamoxifen and maintained biochemical serum parameters, with no change in the metabolism of tamoxifen in the rat. Front. Pharmacol..

